# EHD Proteins Cooperate to Generate Caveolar Clusters and to Maintain Caveolae during Repeated Mechanical Stress

**DOI:** 10.1016/j.cub.2017.07.047

**Published:** 2017-10-09

**Authors:** Ivana Yeow, Gillian Howard, Jessica Chadwick, Carolina Mendoza-Topaz, Carsten G. Hansen, Benjamin J. Nichols, Elena Shvets

**Affiliations:** 1 MRC-Laboratory of Molecular Biology, Francis Crick Avenue, Cambridge CB2 2QH, UK

**Keywords:** caveolae, caveolin, cavin, EHD2, membrane, stretch, cell

## Abstract

Caveolae introduce flask-shaped convolutions into the plasma membrane and help to protect the plasma membrane from damage under stretch forces. The protein components that form the bulb of caveolae are increasingly well characterized, but less is known about the contribution of proteins that localize to the constricted neck. Here we make extensive use of multiple CRISPR/Cas9-generated gene knockout and knockin cell lines to investigate the role of Eps15 Homology Domain (EHD) proteins at the neck of caveolae. We show that EHD1, EHD2, and EHD4 are recruited to caveolae. Recruitment of the other EHDs increases markedly when EHD2, which has been previously detected at caveolae, is absent. Construction of knockout cell lines lacking EHDs 1, 2, and 4 confirms this apparent functional redundancy. Two striking sets of phenotypes are observed in *EHD1,2,4* knockout cells: (1) the characteristic clustering of caveolae into higher-order assemblies is absent; and (2) when the *EHD1,2,4* knockout cells are subjected to prolonged cycles of stretch forces, caveolae are destabilized and the plasma membrane is prone to rupture. Our data identify the first molecular components that act to cluster caveolae into a membrane ultrastructure with the potential to extend stretch-buffering capacity and support a revised model for the function of EHDs at the caveolar neck.

## Highlights

• EHD1, 2, and 4 are all recruited to caveolae
• EHD proteins promote the formation of clustered arrays of caveolae
• EHD proteins are required for morphogenesis of caveolae under repeated stretch forces
• Loss of EHD proteins increases stretch-induced damage to the plasma membrane

## Teaser

Yeow et al. apply gene-editing approaches to show that members of the EHD protein family are recruited to plasma membrane invaginations termed caveolae. The EHD proteins act to promote clustering of caveolae into higher-order assemblies and also to protect the plasma membrane from rupture under stretch forces.

## Introduction

Caveolae are flask-shaped invaginations of the plasma membrane. They are especially abundant in endothelial cells, adipocytes, and muscle cells [1, 2]. The phenotypes of mice lacking key components of caveolae, and of human patients with rare mutations, show that caveolae are important for the maintenance of the normal physiological function in these cell types [1].

Caveolae protect cells from rupture of the plasma membrane under mechanical stress [3, 4]. When tension in the plasma membrane increases, caveolae disassemble or flatten out, and it is possible that the consequent release of membrane convolution may act as a buffer to prevent excessive tension from breaking the membrane [4, 5, 6, 7]. Further activities of caveolae in sensing mechanical force and transducing consequent intracellular signals are also likely to be significant [3, 8], and caveolae may also help maintain membrane integrity through endocytosis of damaged membrane regions [9, 10]. Caveolae have been associated with a range of additional functions, including signal transduction, lipid homeostasis, and endocytosis [8, 11, 12, 13, 14, 15, 16, 17].

Given the potential importance of caveolae as reservoirs of membrane, it is notable that caveolae not only generate membrane convolution because of their individual morphology but also associate to form extensive higher-order clusters of inter-linked caveolae [18, 19, 20]. One reason why the functional relevance of caveolar clusters remains incompletely understood is that the molecular mechanism for linking multiple caveolae together has been unclear.

The protein complex that shapes the caveolar bulb is composed of caveolin and cavin proteins [21, 22]. In mammals there are three caveolins, caveolin1 being essential for generating morphologically defined caveolae in non-muscle cells [23, 24]. There are four cavins, cavin1 (also termed PTRF [25, 26, 27]) being the most important. Cavin1 is essential for producing caveolae in all cell types [25]. The precise stoichiometry with which cavins and caveolins associate means that studies overexpressing individual components are prone to artifact and are hard to interpret, and even moderate overexpression of caveolin1-GFP generates altered sub-cellular dynamics [22, 28, 29]. Gene editing to insert fluorescent protein tags into proteins expressed from endogenous gene loci offers a solution to this problem [30, 31].

Protein complexes at the neck of caveolae are less well characterized than those at the bulb. They contain EHD2 and may contain pacsin2 (syndapin2) and dynamin2 at specific points in the dynamic life cycle of individual caveolae [32, 33, 34, 35]. EHD2 is a member of the Eps15 Homology Domain (EHD) proteins, of which there are 4 in mammals [36]. Despite their high level of amino acid sequence identity (70%–86%), EHDs have been reported to have different localizations and functions. Only EHD2 has been conclusively detected at caveolae [32, 33, 34], and it is also unique among EHDs in having a nuclear localization signal and a potential role in transcriptional regulation within the nucleus [37]. EHD1 and EHD3 have overlapping but distinct roles in the dynamics of peri-nuclear tubular endosomal membranes [38, 39, 40, 41, 42, 43, 44]. EHD4 is also involved in endosomal membrane dynamics [45]. EHD1, EHD3, and EHD4 have been shown to bind to each other [36]. EHDs 1 and 3 have also been shown to act together in vesicle trafficking steps critical for early events in the establishment of a primary cilium [38, 40].

The ATPase activity of EHD2 drives membrane constriction and remodeling in vitro, behavior analogous to that of the related mechano-enzyme dynamin [46]. Dynamin has a well-established direct role in membrane scission and vesicle budding [47]. However, experiments in cells show that loss of EHD2 expression or reduced EHD2 ATPase activity caused increased dynamics of caveolin1, leading to the interpretation that EHD2 acts to stabilize caveolae at the plasma membrane [32, 33]. This posits the role of EHD2 in membrane dynamics as being opposite to that of dynamin, in that guanosine triphosphate (GTP) hydrolysis by dynamin drives membrane shape changes coupled to scission of vesicles while ATP hydrolysis by EHD2 is thought to drive membrane shape changes and stabilize caveolae.

The current study was initiated with the goal of ascertaining more about how EHD2 activity is linked to the dynamics or budding of caveolae. We found, however, that in cells where the *EHD2* gene is effectively deleted there are minimal effects on caveolar dynamics. Further experiments revealed that this is due to functional compensation by *EHD1* and *EHD4*. Cells lacking all of the members of the EHD family showed caveolar phenotypes that are not observed in the *EHD2* knockout cells and provide new insight into the function of EHDs at caveolae.

## Results

### Minimal Effects on the Abundance, Dynamics, and Sub-cellular Distribution of Caveolae in *ΔEHD2* Cells

We used CRISPR/Cas9 to generate NIH 3T3 cells where mutations in *EHD2* lead to the loss of expressed protein (*ΔEHD2*; Figure S1A). Quantification of the number of morphologically defined caveolae in these cells did not reveal a loss of caveolae from the plasma membrane (Figure S1B). We also generated NIH 3T3 cells lacking caveolin1 (*ΔCAV1*) to act as a positive control, and here the loss of caveolae was readily detected (Figure S1B).

In order to assay dynamics of caveolae in *ΔEHD2* cells, CRISPR/Cas9 and an appropriate targeting construct were used to express GFP fused to the C terminus of endogenous caveolin1. Fluorescence recovery after photobleaching (FRAP) experiments on these cells, and control NIH 3T3 cells where endogenous caveolin1 had been tagged in the same way [30], did not detect altered mobility of caveolin1-GFP in the *ΔEHD2* cells (Figure S1C).

Surface biotinylation with NHS-SS-Biotin, followed by selective removal of extracellular biotin, was used to specifically label all endocytic compartments [48]. The proportion of endogenously tagged caveolin1-GFP co-localizing with endocytic compartments appeared the same in *ΔEHD2* and control cells (Figure S1D).

The lack of clear effects on caveolar abundance, dynamics, and sub-cellular distribution in *ΔEHD2* cells contrasts with increased internalization or dynamics of caveolin1 reported when EHD2 is knocked down using small interfering RNAs (siRNAs) [32, 33]. We and others have noted some variable and limited co-localization between tagged and overexpressed EHD1, EHD3, or EHD4 and caveolar markers [32, 34]. This suggested that the activity of other EHD proteins at caveolae could be relevant to the mild phenotypes of *ΔEHD2* cells.

### EHD1 and EHD4 Are Recruited to Caveolae

We produced NIH 3T3 cells expressing GFP fused at the C terminus of endogenous EHD1, EHD2, and EHD4 using CRISPR/Cas9 (Figure S2). The same approach did not yield detectable expression of tagged EHD3. PCR on cDNA from NIH 3T3 cells did not reveal the expression of EHD3. We therefore presumed that EHD3 was not expressed in our cells. Unless otherwise stated, all further experiments in this study used EHD proteins and caveolar markers (caveolin1 and cavin1) fused to fluorescent proteins expressed from their endogenous genomic loci in NIH 3T3 cells, and, for simplicity, we refer to them simply as the expressed fusion protein (“EHD2-GFP,” and so on).

**Figure 1:**
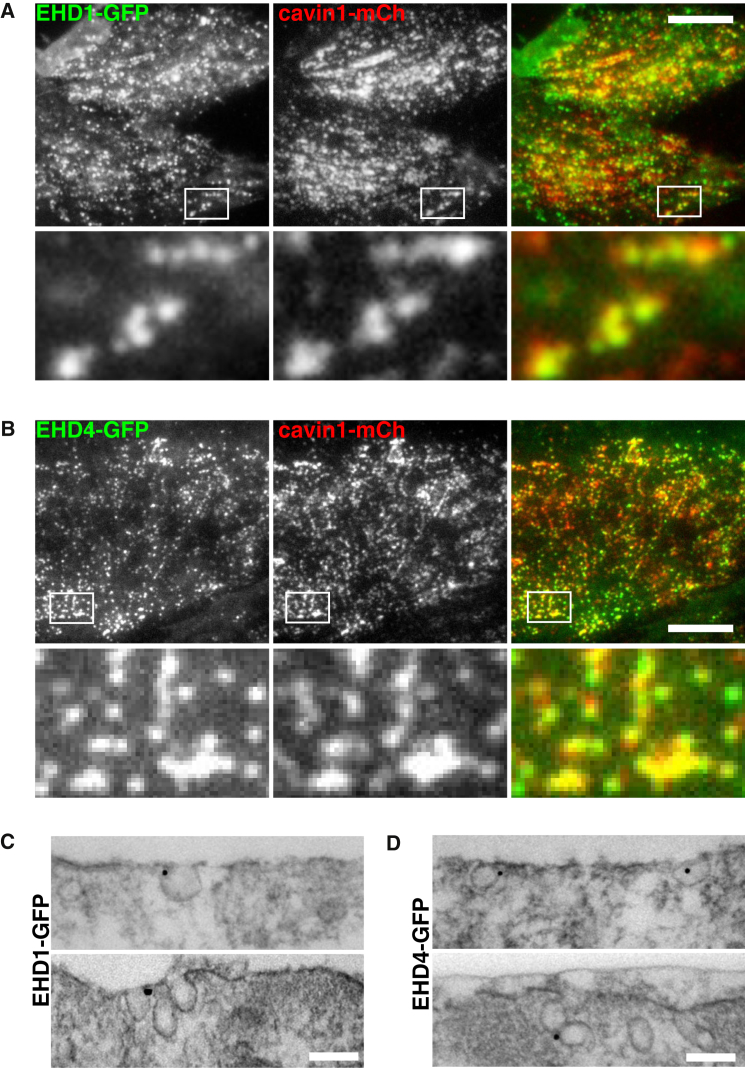
EHD1-GFP and EHD4-GFP Are Present in Caveolae When Expressed at Endogenous Levels (A) TIR imaging of EHD1-GFP and cavin1-mCherry expressed by gene editing in live NIH 3T3 cells. Scale bar, 10 μm. (B) TIR imaging of EHD4-GFP and cavin1-mCherry expressed by gene editing in live NIH 3T3 cells. Scale bar, 10 μm. (C) Immunoelectron microscopy with anti-GFP antibodies in cells expressing EHD1-GFP by gene editing. Scale bar, 100 nm. (D) Immunoelectron microscopy with anti-GFP antibodies in cells expressing EHD4-GFP by gene editing. Scale bar, 100 nm. See also Figures S1–S3.

EHD2-GFP, as predicted, co-localized with the caveolar marker cavin1-mCherry [32, 33, 34]. EHD1-GFP and EHD4-GFP had the punctate distribution previously described for these proteins, partially co-localized with endocytosed transferrin, and they were also present in linear tube-like structures [43, 45, 49] (Figure S3). Total internal reflection (TIR) microscopy, however, revealed smaller structures containing both proteins closely associated with the plasma membrane, and these frequently co-localized with cavin1-mCherry (Figures 1A and 1B). Therefore, a fraction of the total EHD1-GFP and EHD4-GFP expressed is likely to be recruited to caveolae. Use of a pixel mask-based quantitative approach allowed us to estimate that over 90% of EHD2-GFP detected in TIR images is in caveolae, while for both EHD1-GFP and EHD4-GFP the proportion is around 30%.

Immunoelectron microscopy of EHD1-GFP and EHD4-GFP using pre-embedding labeling with affinity-purified anti-GFP antibodies confirmed that both proteins can be detected in caveolae (Figures 1C and 1D). No specific labeling with these antibodies was detected in cells that do not express GFP. Gold particles were frequently detected at the neck of caveolae, agreeing with the apparent distribution of EHD2 around the caveolar neck [22, 32].

### EHD2 Co-precipitates with Both EHD1 and EHD4

**Figure 2:**
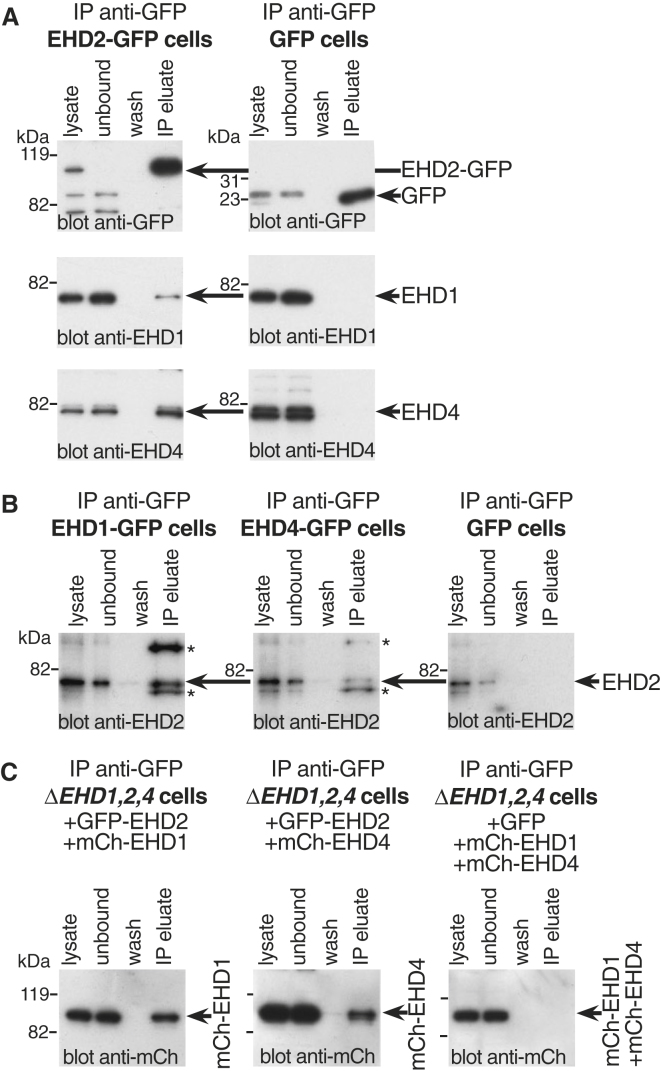
Co-immunoprecipitation of EHD2 with EHD1 and EHD4 (A) Lysates from cells expressing EHD2-GFP by gene editing of the *EHD2* locus or negative controls expressing GFP alone were incubated with anti-GFP antibody beads. The lysate after this incubation is shown as “unbound” and washes from the isolated beads as “wash.” Sample eluted from the beads with sample buffer is shown as “IP eluate” and is concentrated 10× relative to the lysate. (B) Lysates from cells expressing EHD1-GFP by gene editing of the *EHD1* locus, EHD4-GFP by gene editing of the *EHD4* locus, or negative control cells stably transfected with plasmid to express GFP alone were incubated with anti-GFP antibody beads as in (A). The eluate was concentrated 50× relative to the lysate. Anti-EHD2 antibodies cross-react with EHD1 and EHD4; the cross-reacting bands are indicated with an asterisk and the EHD2 band is arrowed. (C) Lysates from *ΔEHD1,2,4* triple-knockout NIH 3T3 cells transiently transfected with EHD-expressing or GFP-expressing plasmids as shown were incubated with anti-GFP antibody beads. The eluate was concentrated 10× relative to the lysate.

The presence of EHD1 and EHD4 in caveolae at the same time as EHD2 suggested that they may form heteromeric complexes. EHD2-GFP could be efficiently precipitated with anti-GFP antibodies. This resulted in specific co-precipitaton of EHD1 and EHD4 (Figure 2A). We also carried out the reverse immunoprecipitation, precipitating EHD1-GFP or EHD4-GFP and blotting for endogenous EHD2 (Figure 2B). Again, a fraction of total EHD2 was specifically associated with EHD1 and EHD4.

These data suggest that EHD2 can form complexes containing both EHD1 and EHD4. To confirm this and to ask whether these complexes require the presence of both EHD1 and EHD4 at the same time, we used transient transfection to express GFP-EHD2 and either mCherry-EHD1 or mCherry-EHD4 in a cell line lacking EHD1, EHD2, and EHD4 (*ΔEHD1,2,4* described below). Specific association between EHD2 and EHD1 or EHD4 was detected, and the formation of the relevant complexes was not contingent on the presence of all three EHD proteins (Figure 2C).

### EHD1 Recruitment to Caveolae Is Increased in *ΔEHD2* Cells

**Figure 3:**
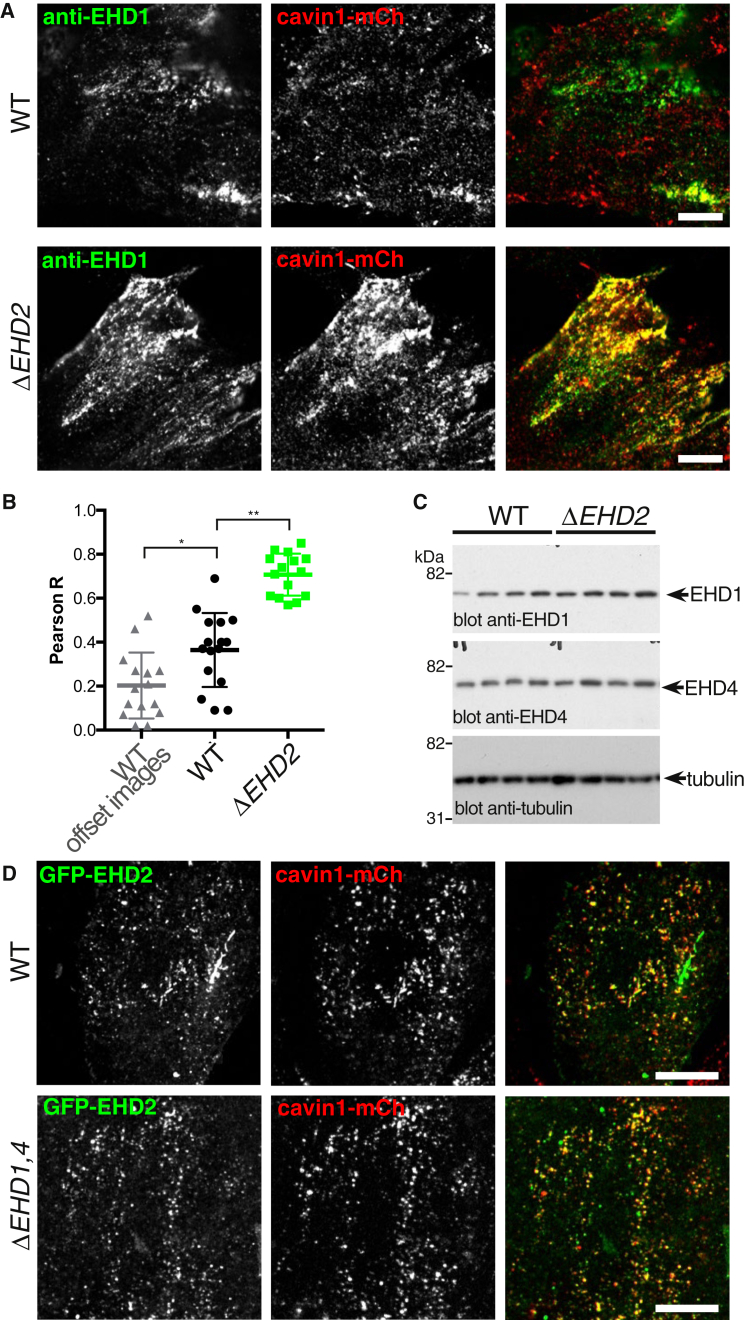
Recruitment of EHD1 to Caveolae Is Significantly Increased in Δ*EHD2* Cells (A) Confocal microscopy of fixed wild-type (WT) and Δ*EHD2* NIH 3T3 cells, both expressing cavin1-mCherry from the endogenous locus. Cells were labeled with anti-EHD1 antibodies for indirect immunofluorescence. Scale bar, 10 μm. See also Figures S1 and S4. (B) Quantification of co-localization between EHD1 and cavin1-mCherry using Pearson’s correlation coefficient R. Each data point represents one cell region. The WT images were analyzed with the two fluorescence channels offset by ∼0.5 μm to give an indication of the values expected due to chance overlap. Student’s t test, ^∗^p < 0.05 and ^∗∗^p < 0.01. (C) Western blots to show the abundance of EHD1 and EHD4 proteins in *ΔEHD2* cells. Blots from four cultures of WT NIH 3T3 cells and four clones of *ΔEHD2* cells derived from them are shown. (D) Confocal images showing co-localization between GFP-EHD2 expressed by transient transfection and cavin1-mCherry expressed from the endogenous locus, in control WT NIH 3T3 cells and in *ΔEHD1,4* NIH 3T3 cells that do not express EHD1 or EHD4. Scale bars, 10 μm. See also Figure S5.

We produced cell lines lacking EHD1 and EHD4, once more using CRISPR/Cas9 (*ΔEHD1* and *ΔEHD4*; Figures S2 and S4) and identified an antibody against EHD1 that provides highly specific staining via indirect immunofluorescence (Figure S2). Control NIH 3T3 cells expressing cavin1-mCherry and *ΔEHD2* cells also expressing cavin1-mCherry were labeled with the anti-EHD1 antibody (Figure 3A). Confocal images revealed a marked redistribution of EHD1 in the knockout cells, with much more co-localization with cavin1-mCherry apparent. Indeed, in the *ΔEHD2* cells, EHD1 co-localized with cavin1-mCherry to an extent indistinguishable from the co-localization exhibited between EHD2-GFP and cavin1-mCherry in wild-type (WT) cells (Figure S5). Co-localization between EHD1 and cavin1-mCherry was quantified using Pearson’s correlation coefficient, confirming the increase in *ΔEHD2* cells (Figure 3B). The change in distribution of EHD1 was not accompanied by a significant change in expression levels of this protein or EHD4 (Figure 3C). As EHD1 and EHD4 co-localize extensively and bind to each other, it is likely that EHD4, like EHD1, re-localizes to caveolae in *ΔEHD2* cells [36].

We used CRISPR/Cas9 to produce NIH 3T3 cells lacking expression of both EHD1 and EHD4. Co-localization between GFP-EHD2 expressed by transient transfection and cavin1-mCherry expressed by genome editing was indistinguishable in WT and the *ΔEHD1,4* cells (Figure 3D), so recruitment of EHD2 to caveolae is not dependent on the presence of EHD1 or EHD4. We conclude that there is unlikely to be any co-dependency between EHD1, 2, and 4 for recruitment to caveolae, and that EHD1 and EHD4 could therefore compensate when EHD2 is absent.

### *ΔEHD1,2,4* Cells Lack Higher-Order Clusters of Caveolae

**Figure 4:**
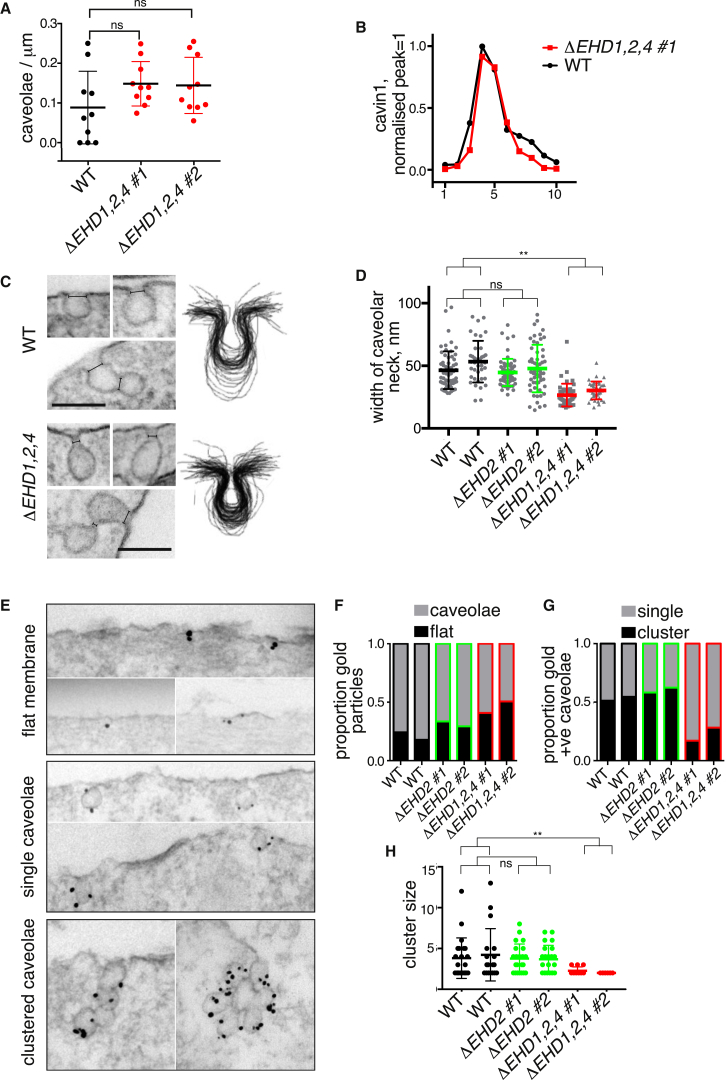
The Ultrastructure of the Caveolar Neck and the Formation of Clustered Arrays of Caveolae Are Dependent on EHD Proteins (A) Quantification of morphologically defined caveolae in *ΔEHD1,2,4* triple-knockout NIH 3T3 cells (Figure S6). Two different clones of *ΔEHD1,2,4* cells were analyzed. Statistical analysis used one-way ANOVA with Dunnett’s multiple comparison test. (B) Sucrose gradient fractionation of lysates from WT and *ΔEHD1,2,4* cells. Gradient fractions were blotted with antibodies against cavin1, the signal from each fraction quantified using densitometry, and values normalized so that the peak intensity = 1. Each data point is a mean from three separate gradients. See also Figure S6B. (C) Electron micrographs showing ultrastructure of the caveolar neck in control WT and *ΔEHD1,2,4* triple-knockout NIH 3T3 cells. The right-hand images present aggregated membrane profiles from 40 individual caveolae of each genotype. Scale bars, 100 nm. (D) Measurement of the width of caveolar neck, as shown in (C), for multiple caveolae from WT, *ΔEHD2*, and *ΔEHD1,2,4* NIH 3T3 cells. Statistical comparison is one-way ANOVA with Dunnett’s multiple comparison test. (E) Immunoelectron microscopy with anti-caveolin1 antibodies to classify membrane morphology of caveolin1-positive regions. Examples of regions classified as flat membrane, single caveolae, and clustered caveolae are shown. (F) Quantification of the ratio between caveolin1-positive regions classified as flat or as morphological caveolae (clustered + single caveolae) from immunoelectron microscopy as in (E). (G) Quantification of the ratio between caveolin1-positive caveolae classified as single or as in clusters from immunoelectron microscopy. Imaging and quantification are as in (E) and (F). (H) Quantification of the size of caveolar clusters (the number of caveolar bulbs present in a single structure) identified in immunoelectron microscopy as in (C). Statistical comparison is one-way ANOVA with Dunnett’s multiple comparison test, using aggregated data from both samples/clones of each genotype.

To test the hypothesis that EHD1 and 4 compensate for a lack of EHD2 function, we used CRISPR/Cas9 to produce NIH 3T3 cells lacking expression of all three proteins (Figure S6A). The number of caveolae, identified morphologically, was not significantly different between control and *ΔEHD1,2,4* cells (Figure 4A). Consistent with this, cavin proteins were still found in characteristic high-molecular-weight complexes when lysates from *ΔEHD1,2,4* cells were analyzed on sucrose gradients (Figure 4B), and co-localization between gene-edited caveolin1-GFP and anti-cavin1 antibodies was indistinguishable in control and *ΔEHD1,2,4* cells (Figure S6B). EHD proteins are not, therefore, an essential part of the core machinery required for generating the caveolar bulb.

EHD proteins are likely to localize to the neck of caveolae (Figures 1C and 1D) [22, 32, 33]. We asked whether the ultrastructure of the caveolar neck is altered in the *ΔEHD1,2,4* cells. Both inspection of individual caveolae and superimposition of membrane profiles from multiple caveolae showed that the neck of caveolae lacking EHD proteins is constricted when compared to the WT situation (Figure 4C). Measurement of the diameter of the caveolar neck confirmed this (Figure 4D). This phenotype was not observed in cells lacking only EHD2 (Figure 4D).

We used immunoelectron microscopy to carry out a detailed analysis of the morphology of all caveolin1-positive structures in *ΔEHD1,2,4* cells, as well as *ΔEHD2* cells. Caveolin1-positive membrane structures were classified as flat membrane, single caveolae, or clusters of caveolae (Figure 4E). Both *ΔEHD2* and *ΔEHD1,2,4* cells exhibited a slight increase in the amount of caveolin1 found on flat regions of membrane (Figure 4F). Clusters of two or more caveolae were strikingly reduced in the *ΔEHD1,2,4* cells, but not in *ΔEHD2* cells (Figure 4G). Moreover, when the data were analyzed in terms of the size of clusters of caveolae in the different cell lines, it was apparent that there was a loss of clusters of more than three caveolae in the *ΔEHD1,2,4* cells (Figure 4H).

**Figure 5:**
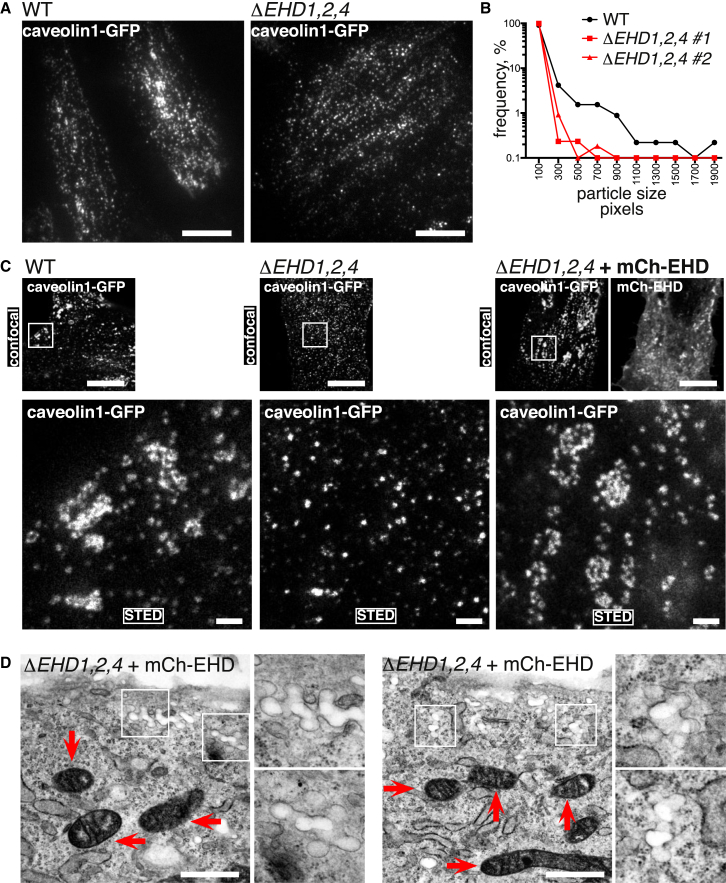
Caveolin1-GFP Clusters Are Generated by EHD Proteins (A) TIR microscopy to show the distribution of caveolin1-GFP in gene-edited WT and *ΔEHD1,2,4* NIH 3T3 cells. Scale bar, 10 μm. (B) Quantification of puncta size in TIR images as shown in (A), shown as frequency distribution of all sizes detected in 10 cells of each genotype shown. (C) Confocal and stimulated emission depletion (STED) microscopy of WT cells, *ΔEHD1,2,4* cells, and *ΔEHD1,2,4* cells expressing mCherry-EHD1, mCherry-EHD2, and mCherry-EHD4 by transient transfection. All cells are gene edited to express caveolin1-GFP. The STED images are of the boxed region in the confocal images. Scale bars, 5 μm (in confocal images) and 1 μm (in STED images). (D) Electron micrographs of *ΔEHD1,2,4* cells expressing mitochondrially targeted APEX, mCherry-EHD1, mCherry-EHD2, and mCherry-EHD4 by transient transfection. Cells were stained with diaminobenzidine, producing electron-dense deposits in the mitochondria of transfected cells. Two cells are shown; arrows highlight mitochondria, and the boxed regions are shown at higher magnification in the additional panels. Scale bars, 500 nm.

We sought ways to confirm the conclusion that EHD proteins promote the formation of the characteristic clusters formed by caveolae in cells. Clustering of caveolae will result in larger or brighter puncta detected by conventional light microscopy. Accordingly, we examined TIR images of caveolin1-GFP expressed in WT and *ΔEHD1,2,4* cells. These confirmed that larger puncta were visibly more scarce in the knockout cells (Figure 5A). Image analysis software was used to measure the size of caveolin1-GFP puncta revealed by TIR illumination in the different cell lines. This confirmed that in WT cells the population of caveolin1-GFP puncta contained a significant number of larger/brighter structures (Figure 5B).

The inference that larger caveolin1-GFP puncta represent clusters of individual caveolae in WT cells was confirmed using stimulated emission depletion (STED) microscopy, as structures detected as larger puncta by conventional confocal imaging could be resolved by STED microscopy into clusters of individual structures, each with the approximate dimensions of the caveolar bulb (Figure 5C). Clusters comprising multiple caveolae were readily detected in WT cells but were much less prominent in *ΔEHD1,2,4* cells (Figure 5C). Importantly, when *ΔEHD1,2,4* cells were transiently transfected with mCherry-EHD1, mCherry-EHD2, and mCherry-EHD4, the presence of clusters comprising multiple caveolin1-GFP puncta was restored.

We used the same approach, re-expressing mCherry-EHD1, mCherry-EHD2, and mCherry-EHD4 by transient transfection in *ΔEHD1,2,4* cells and examined the cells using electron microscopy. The transfection also included mitochondrially targeted APEX, allowing the unambiguous identification of transfected cells through the presence of electron-dense polymerized diaminobenzidine staining within the mitochondrial matrix [50]. In transfected cells, clusters or rosettes of multiple caveolae were readily detected, while these were not observed in untransfected cell populations (Figures 5D and 4H). All of these data reinforce the conclusion that EHD proteins provide a key molecular component for the formation of higher-order clusters of caveolae.

### Turnover and Dynamics of Caveolin1 Are Increased in *ΔEHD1,2,4* Cells

**Figure 6:**
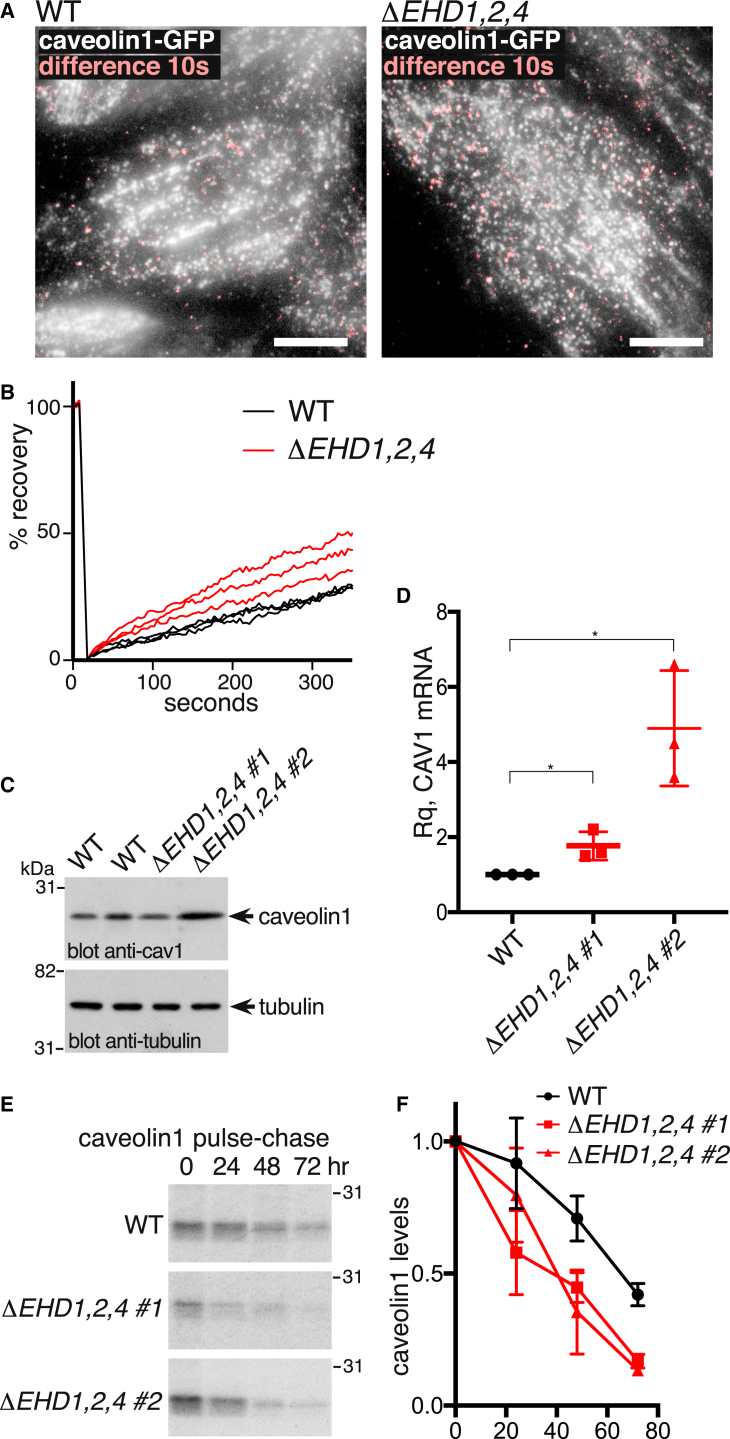
Increased Dynamics and Turnover of Caveolin1 in *ΔEHD1,2,4* Triple-Knockout Cells (A) Averaged projections of the first 10 s of time-lapse TIR microscopy, with the difference between these projections and projections of the next 10 s of the time-lapse sequence overlaid in pink. Original images acquired at 1 Hz. Scale bar, 10 μm. See also Movie S1. (B) Quantification of mobility of caveolin1-GFP by FRAP. Each line is a mean from >7 individual photobleached regions from different experiments. (C) Western blots to show caveolin1 levels in *ΔEHD1,2,4* triple-knockout NIH 3T3 cells. (D) Quantitative measurements of caveolin1 mRNA levels in *ΔEHD1,2,4* triple-knockout NIH 3T3 cells, using real-time PCR. Each point is a separate biological replicate itself based on four experimental replicates. Normalization was to GAPDH. Statistical analysis is one-way ANOVA with Dunnett’s multiple comparison test. (E) Pulse-chase analysis of caveolin1 turnover. Cells with the genotypes shown were pulsed with ^35^S Methionine, and, after the times indicated, were lysed before immunoprecipitation of caveolin1 and analysis by SDS-PAGE and autoradiography. (F) Quantification of pulse-chase experiments as in (E) using densitometry of autoradiograms (n = 3).

Previous experiments have shown that siRNA-mediated knockdown of EHD2 results in increased mobility of caveolin1-GFP puncta [32, 33]. We asked whether the same phenomenon is detected in our *ΔEHD1,2,4* cells. Time-lapse TIR imaging revealed mobile caveolin1-GFP puncta in both control and *ΔEHD1,2,4* cells, with more appearing in the latter (Figure 6A; Movie S1). FRAP experiments confirmed that caveolin1-GFP is more mobile in the *ΔEHD1,2,4* cells than in controls (Figure 6B).

Increased mobility of caveolin1-GFP in *ΔEHD1,2,4* cells could reflect the destabilization of caveolae in the absence of EHD proteins. We asked whether turnover of caveolin1 is increased in the *ΔEHD1,2,4* cells. Although *ΔEHD1,2,4* clones did not exhibit consistent changes in the steady-state levels of caveolin1 protein (Figure 6C), in all cases qPCR showed a significant increase in *CAV1* mRNA abundance (Figure 6D). As these observations predict, ^35^S Methionine pulse-chase analysis revealed that caveolin1 was consistently degraded more quickly in the *ΔEHD1,2,4* cells (Figures 6E and 6F).

### EHD Proteins Protect Cells from Repeated Mechanical Stress

**Figure 7:**
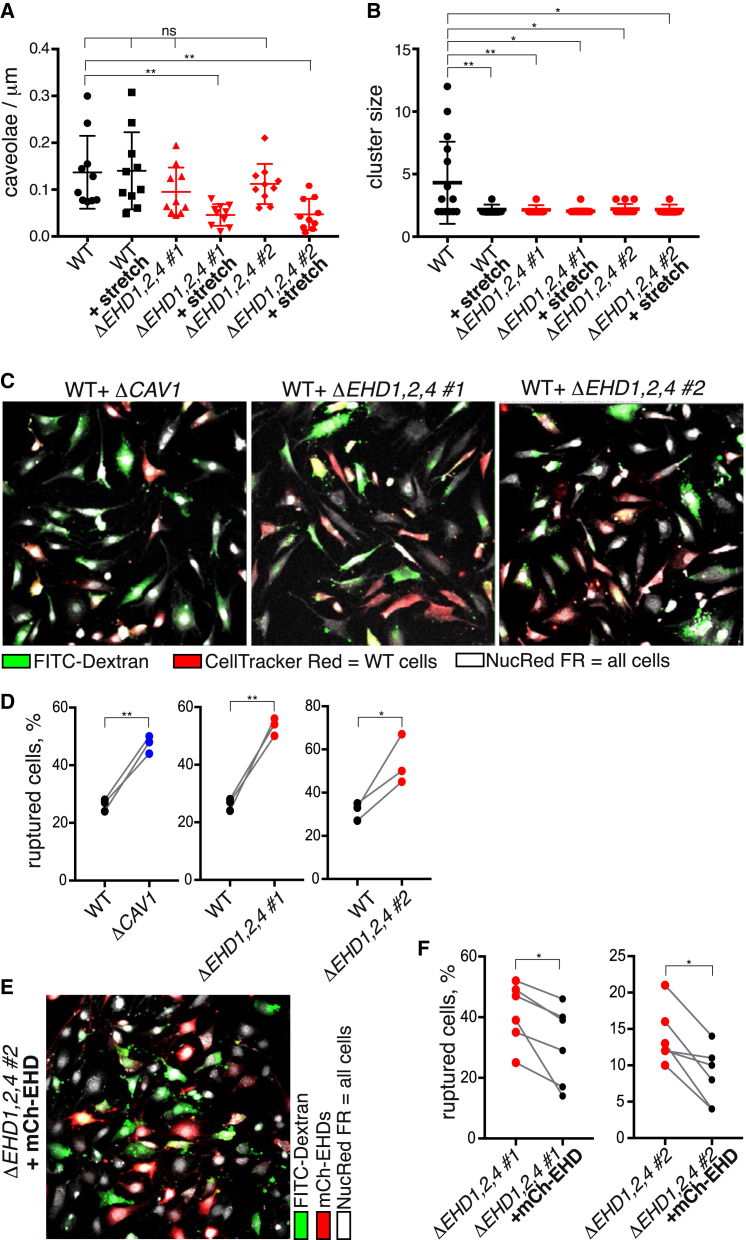
EHD Proteins Are Required for Caveolar Stability under Repeated Mechanical Stress, and Cells Lacking EHD Proteins Are More Likely to Rupture under Repeated Mechanical Stress (A) Quantification of morphologically defined caveolae in *ΔEHD1,2,4* triple-knockout NIH 3T3 cells by electron microscopy. Cells were grown on deformable silicon substrate and stretched by 20% at 1.5 Hz for 60 min as indicated. For each genotype, complete reconstructions of the perimeter of 10 cells were generated from 15–70 high-resolution micrographs per cell. Statistical analysis used one-way ANOVA with Dunnett’s multiple comparison. Also see Figure S7. (B) Quantification of the sizes of clusters of caveolae from immunoelectron microscopy as in Figure 4E, but with cells fixed during repetitive stretching. For each genotype shown, 50 micrographs of regions selected as containing positive staining were acquired at 6,500× magnification. Statistical analysis used one-way ANOVA with Dunnett’s multiple comparison test. (C) Assay for plasma membrane rupture in cells with genotypes as shown. Rupture is indicated by cytoplasmic accumulation of 150 kDa fluorescein isothiocyanate (FITC)-dextran (green). Cells were stretched for 60 min at 1.5 Hz. White signal is from NucRed Live 647 dye. WT cells were labeled with Cell Tracker red. (D) Quantification of the incidence of plasma membrane rupture as in (C) above, expressed as the proportion of the total number of cells that have green cytoplasmic staining. Each point represents 6–10 images from a single experiment, each image containing 50–150 individual cells. The data from each experiment are paired to allow comparison of cells with different genotypes grown in the same mixed population. Statistical analysis is by paired t test. (E) Assay for plasma membrane rupture in *ΔEHD1,2,4* triple-knockout NIH 3T3 cells, some of which are transiently transfected with mCherry-EHD1, mCherry-EHD2, and mCherry-EHD4. White signal is from NucRed Live 647 dye. (F) Quantification of the incidence of plasma membrane rupture in transfected versus non-transfected *ΔEHD1,2,4* triple-knockout cells as in (E). Statistical analysis is by paired t test.

Previous structural and biochemical experiments showed that EHDs act to deform or sculpt membranes [46, 51]. This property could be important for the kinetics of re-formation of caveolae after they have flattened out under mechanical stress [4]. Cells were grown on a deformable substrate and subjected to cycles of stretching by 20% at 1.5 Hz for 60 min. The number of morphologically defined caveolae was markedly decreased in *ΔEHD1,2,4* cells under these conditions (Figure 7A). EHDs are, therefore, indeed required to maintain steady-state levels of caveolae when cells are repeatedly stretched, and the caveolae present in *ΔEHD1,2,4* cells are not equivalent to caveolae in WT cells. Quantification of the abundance of clusters of multiple caveolae under stretching conditions confirmed the absence of clusters in the *ΔEHD1,2,4* cells and revealed that, even in WT cells, stretching decreased the number of clusters (Figure 7B).

Caveolae protect cells from membrane damage when cells are under mechanical stress [4, 5, 6]. We assayed transient loss of plasma membrane integrity by incubating cells in high concentrations of fluorescent dextran. If plasma membrane barrier function is lost under these conditions, fluorescent dextran will gain access to the cytosol [52, 53]. Cells grown on a deformable substrate were subjected to cycles of stretching by 20% at 1.5 Hz for 60 min. There was no change in expression of caveolin1 or other caveolar components during the time span of these experiments (Figure S7). The likelihood of membrane rupture in this experimental system depends critically on the density of cells and potentially on further local factors. In order to mitigate such factors, cells of different genotypes were plated as mixed populations, after labeling with Cell Tracker dyes to allow discrimination between genotypes (Figure 7C). Quantitative comparisons were then made between cells growing as a single mixed population. There was a significant increase in the proportion of cells containing cytosolic fluorescent dextran in both *ΔCAV1* and *ΔEHD1,2,4* cells (Figures 7C and 7D), suggesting that EHDs, like caveolin1, help to protect cells from damage under mechanical stress.

To obtain more direct evidence that the increased propensity for membrane rupture exhibited by *ΔEHD1,2,4* cells is directly attributable to a lack of the EHD proteins, these cells were transfected with mCherry-tagged EHD1, EHD2, and EHD4 (Figure 7E) and subjected to stretching in the presence of fluorescent dextran as above. Cells transfected with the EHDs were more likely to exclude fluorescent dextran than neighboring untransfected cells (Figures 7E and 7F). This confirms that EHD proteins help to maintain the integrity of the plasma membrane under exposure to stretch forces.

## Discussion

Our data provide three key sets of new observations germane to the function of EHD proteins at caveolae. (1) We show that EHD1 and EHD4 can be detected in caveolae, that the amount of EHD1 present in caveolae increases when EHD2 is deleted, and that the absence of all three of these EHD proteins is required to produce detectable effects on the dynamics and distribution of caveolin1. (2) We show that characteristic clusters of multiple caveolae are dependent on the presence of all three EHD proteins. (3) We show that in cells lacking all three EHD proteins, repeated mechanical stress results in the loss of morphologically-defined caveolae and an increased likelihood of plasma membrane rupture.

Previous experiments show that EHD2 is present at the neck of caveolae, and is not part of the large caveolar coat complex that shapes the caveolar bulb [22, 32]. Our new data showing that EHD1 and EHD4 bind to EHD2, and that immuno-electron microscopy against EHD1 and EHD4 results in labeling close to the neck, suggest that EHD1 and EHD4 are also found at the neck domain. We can now state that the loss of EHD proteins has no discernable effect on the shape of the caveolar bulb, but does result in ultrastructural changes to the shape of the neck region.

The structural and biochemical properties of EHD2 are understood in some detail and reveal EHDs as mechano-enzymes that utilize energy released by ATP hydrolysis to drive changes in membrane shape [46, 51, 54]. These properties, coupled with our data showing that the abundance of caveolae is only dependent on EHDs when cells are under repeated mechanical stress, suggest to us that one contribution of EHDs to the functional properties of caveolae is to promote rapid re-formation after mechanically induced flattening, by facilitating membrane curvature.

The absence of membrane convolutions introduced by caveolae correlates with an increased likelihood that the plasma membrane will undergo damage under mechanical stress [4, 5, 6]. Our data extend this correlation. We show that *ΔEHD1,2,4* cells lack the convolutions introduced by higher-order clusters of caveolae, that *ΔEHD1,2,4* cells under conditions of repeated stretching have a significant reduction in the total number of caveolae, and that *ΔEHD1,2,4* cells are more likely to suffer stretch-induced membrane damage. These observations support a model in which the membrane convolutions introduced by caveolae act as a buffer, flattening out under stretch forces and thereby reducing the likelihood of membrane damage. We point out, however, that neither our new experiments nor those in previous publications unambiguously demonstrate that such a stretch-buffering effect is the main or sole cause of the mechano-protective activity of caveolae. It is possible that signaling from caveolae contributes to mechano-protection, and it is possible that functions of the EHDs outside of caveolae, for example, in endocytic processes, are also relevant.

We demonstrate that EHD1 and EHD4 can compensate for the lack of EHD2 in *ΔEHD2* cells. This evident functional redundancy may well be relevant for other aspects of the cell biology of the EHD proteins. Precisely why multiple EHDs are required at caveolae, or at other functional locations, is not yet clear. It may be that they recruit different binding partners or have subtly different effects on membrane curvature. Additionally, in WT cells only a small fraction of the total EHD1 and EHD4 present is recruited to caveolae, so there must be mechanisms to regulate the recruitment of EHDs to different membrane locations. Our EHD knockout and fluorescent protein knockin cell lines should prove useful reagents for tackling these questions.

## STAR★Methods

### Key Resources Table

**Table:** 

REAGENT or RESOURCE	SOURCE	IDENTIFIER
**Antibodies**		

rabbit anti-cavin1	Abcam	Cat# ab48824
rabbit anti-caveolin1	BD Bioscience	Cat# 610060
mouse anti-GFP	Roche	Cat# 11814460001
rabbit anti-RFP (used to detect mCherry)	MBL International	Cat# PM005
mouse anti-tubulin	Sigma	Cat# T9026
goat anti-EHD2	Abcam	Cat# ab23935
rabbit anti-EHD1	Abcam	Cat# ab109311
rabbit anti-EHD4	Proteintech	Cat# 11382-2-AP
normal goat serum	Aurion	Cat# 905.002
rabbit anti-GFP	Abcam	Cat# ab6556
F(ab’)2 goat anti-rabbit ultrasmall gold	Aurion	Cat# 100.166

**Chemicals, Peptides, and Recombinant Proteins**		

GFP-Trap agarose beads	Chromotek	Cat# gta-10
Protein A Sepharose beads	GE Healthcare	Cat# 17-0780-01
Agarose beads	Chromotek	Cat# bab-20
‘cOmplete’ protease inhibitors	Roche	Cat# 04693159001
R-Gent SE-EM for silver enhancement	Aurion	Cat# 500.033

**Critical Commercial Assays**		

TaqMan Universal Master Mix II	Applied Biosystems	Cat# 4440043
EasyTag EXPRESS ^35^S Protein Labeling Mix	Perkin Elmer	Cat# NEG772002MC
Gibson Master kit	New England Biolabs	Cat# E2611
ECL Western Blot Detection Reagent Kit	GE Healthcare	Cat# RPN2209
Immobilon Western Chemiluminescent HRP Substrate	Millipore	Cat# WBKLS
RNeasy Mini Kit	QIAGEN	Cat# 74104
High-Capacity RNA-to-cDNA Kit	Applied Biosystems	Cat# 4387406

**Experimental Models: Cell Lines**		

NIH 3T3 cells	Harvey McMahon, MRC-LMB	ATCC Cat# CRL-1658

**Oligonucleotides**		

EHD1 PCR genotyping forward CCGTCCTGTAGCAGCCAG	This paper	N/A
EHD1 PCR genotyping reverse CCGTGCATGACCGCGATG	This paper	N/A
EHD2 PCR genotyping forward CTCTCCACCTTGTAGTCTCC	This paper	N/A
EHD2 PCR genotyping reverse CAGGGGAAGAAGTTTCGTGC	This paper	N/A
EHD4 PCR genotyping forward GGTTCTTACTGAAGTGCGGC	This paper	N/A
EHD4 PCR genotyping reverse CCTTGGCAACAGCAAGGAAG	This paper	N/A
All oligonucleotides used for genome editing are shown in Table S1	N/A	N/A

**Recombinant DNA**		

mCherry-EHD1	Carsten Hansen, University of Edinburgh [34]	N/A
mCherry-EHD2	Carsten Hansen, University of Edinburgh [34]	N/A
mCherry-EHD4	Carsten Hansen, University of Edinburgh [34]	N/A
GFP-EHD2	Harvey McMahon, MRC-LMB [46]	N/A
Mito-V5-APEX	Sean Munro, MRC-LMB [50]	Addgene plasmid #42607
pSpCas9(BB)-2A-GFP	Feng Zhang, MIT	Addgene plasmid #48138
pSpCas9(BB)-2A-Puro	Feng Zhang, MIT	Addgene plasmid #48139
piRFP670-N1	Vladislav Verkhusha, Albert Einstein College of Medicine.	Addgene plasmid #45457

**Software and Algorithms**		

Graphpad Prism	Graphpad	https://www.graphpad.com/scientific-software/prism/

**Other**		

ShellPa cell stretching device	Menicon Life Science	Model# NNMS

### Contact for Reagent and Resource Sharing

Requests for further information, resources and reagents should be directed to and will be fulfilled by the Lead Contact, Ben Nichols (ben@mrc-lmb.cam.ac.uk).

### Experimental Model and Subject Details

NIH 3T3 cells (kind gift from the McMahon lab, MRC-LMB) were grown in DMEM (GIBCO) supplemented with penicillin and streptomycin and 10% calf serum at 37°C. Cells were tested for Mycoplasma contamination but were not authenticated.

### Method Details

#### Genome editing

Generation of NIH 3T3 cell lines expressing caveolin1-GFP or/and cavin1-mCherry tagged from endogenous loci were described previously [30]. For tagging or deleting endogenous EHD1, EHD2, and EHD4 proteins, as well as deletion of endogenous Caveolin1/CAV1, Cas9 used in this study was applied as described in [31]. For each cleavage site, two potential guide RNA sequences were designed using Feng Zhang lab software at http://crispr.mit.edu/ (for sequences see Table S1). The chosen nucleotide sequences were used for insertion into pSpCas9(BB)-2A-GFP (PX458) or pSpCas9(BB)-2A-Puro (PX459), that were a gift from Feng Zhang (Addgene plasmid #48138 and #48139).

Donor DNA constructs containing flanking regions for gap repair by homologous recombination and the appropriate fluorescent protein DNA were produced as follows. Approximately 1 kb of genomic DNA sequence on either side of the *EHD1, EHD2* and *EHD4* stop codons were amplified from genomic DNA using primers listed in Table S1. DNAs coding for GFP, mCherry or iRFP [55], originated from pEGFP-N1, pCherry-N1 (Clontech) or piRFP670-N1 (gift from Vladislav Verkhusha (Addgene plasmid #45457)) were amplified using the primers listed in Table S1. In donor constructs the stop codons of the *EHD1, EHD2* or *EHD4* genes were deleted and cDNA of fluorescent protein was fused with linker as described [56], and inserted into pBlueScript SK (-)- using Gibson Master kit assembly (New England BioLabs) according to the manufacturer’s instructions.

For generation of genome-edited NIH 3T3 cell lines with tagged proteins, PX459 plasmids with appropriate guiding RNA sequences and donor plasmids were co-transfected into cells using Neon transfection system (Invitrogen). After transfection, cells were cultured for 5 days to recover and express the protein of interest, and sorted for relevant fluorescent signals using a Sony iCyt Synergy Dual Channel High Speed Cell sorter or Beckman Coulter MoFlo High Speed Cell sorter to obtain populations of positive cells. Genome edited cells expressing tagged proteins were not cloned, but were cultured as populations of positive cells. For generation of genome-edited NIH 3T3 cell lines deleted of the protein of interest, PX458 plasmids with appropriate guiding RNA sequences were transfected into cells using Neon transfection system and sorted for GFP-positive signal. For generation of cells deleted of two/three genes, cells were co-transfected with several PX458 plasmids simultaneously. Cell lines with gene knockouts were cloned, and screened as individual clones. Correct gene targeting was determined by PCR, and by western blotting.

#### Cell surface biotinylation and internalization assay

For the internalization assay, cells seeded on fibronectin were washed twice with PBS pH 7.9 and subsequently cell surface molecules were biotinylated with 0.2 mg/ml sulfo-NHS-SS-biotin in the same buffer at 37°C. The reaction was quenched 15 min later with 50 mM Tris and then surface exposed biotin was removed by incubating the cells for 3 × 7 min in 100 mM MESNA (Sodium 2-mercaptoethanesulfonate) in MESNA buffer (50 mM Tris, 100 mM NaCl, 1 mM EDTA, 0.2% (w/v) BSA, pH 8.6 at 25°C). Cells were fixed with 4% paraformaladehyde in PBS, permeabilized and labeled with Alexa-546 streptavidin (Invitrogen).

#### Polymerase chain reaction

Genomic DNA from cells were extracted using cell lysis and protein precipitation solution (QIAGEN). The gDNA was PCR amplified using KOD Hot Start DNA Polymerase (Novagen). Primers used for amplification of EHD1 were forward primer CCGTCCTGTAGCAGCCAG and reverse primer CCGTGCATGACCGCGATG, for EHD2 forward primer CTCTCCACCTTGTAGTCTCC and reverse primer CAGGGGAAGAAGTTTCGTGC, and for EHD4 forward primer GGTTCTTACTGAAGTGCGGC and reverse primer CCTTGGCAACAGCAAGGAAG.

#### Transferrin uptake

Cells were incubated with 5 μg/ml transferrin conjugated to Alexa Fluor 647 (Invitrogen) in serum-free media for 30 min at 37°C. Cells were subsequently stained with appropriate antibodies.

#### DNA constructs and transient transfection

Different combinations of mCherry-EHD1, mCherry-EHD2, mCherry-EHD4, GFP-EHD2 (kind gift from the McMahon lab, MRC-LMB), GFP empty, and Mito-V5-APEX (kind gift from the Munro lab, MRC-LMB (Addgene plasmid #42607)) plasmids were singly or co- transiently transfected into the appropriate cells using the Neon transfection system. Cells were cultured for 24 hr and experiments conducted after.

#### Immunoprecipitation

Cells were washed with ice-cold PBS and lysed with IP lysis buffer (50 mM Tris-HCl pH 7.4, 300 mM NaCl, 5 mM EDTA and 0.5% (v/v) Triton X-100, supplemented with ‘cOmplete’ protease inhibitors (Roche)). The lysates were centrifuged at 50,000 rpm for 30 min at 4°C. The supernatants were pre-cleared for 1 hr at 4°C with agarose beads (Chromotek), then incubated with GFP-Trap agarose beads (Chromotek) for 1 hr at 4°C. Proteins were eluted from the beads with sample buffer for 10 min at 95°C. Samples were subjected to western blotting.

For pulse chase samples, cells were washed with ice-cold PBS and lysed with IP lysis buffer (20 mM Tris-HCl pH 7.4, 100 mM NaCl, 5 mM EDTA, 1% Triton X-100 and 1% (w/v) octyl-glucoside, supplemented with protease inhibitors). The lysates were centrifuged at 20,000 g for 20 min at 4°C. The supernatants were pre-cleared for 1 hr at 4°C with Protein A Sepharose beads (GE Healthcare), then incubated with Protein A Sepharose beads and rabbit anti-caveolin1 antibody overnight at 4°C. Proteins were eluted from the beads with sample buffer for 10 min at 95°C.

#### Western blots

Samples were lysed in sample buffer (Novex), boiled and run on pre-cast 4%–20% Tris-Glycine gels (Invitrogen). The gels were then blotted using wet transfer, the membrane blocked in a PBS solution containing 5% dried skimmed milk powder, incubated with the appropriate primary antibodies, washed and incubated with HRP conjugated secondary antibodies. The blots were developed using Immobilon Western Chemiluminescent HRP Substrate (Millipore) or ECL Western Blot Detection Reagent Kit (GE Healthcare) onto Fuji Super RX X-ray films.

#### Sucrose velocity gradients

We used a protocol described in [30]. One Petri dish of each cell line was washed in PBS and solubilized in 1 ml lysis buffer (20 mM Tris-HCl pH 8, 100 mM NaCl, 5 mM EDTA and 0.5% Triton X-100, supplemented with protease inhibitors). The samples were next incubated for 20 min at RT. Post-nuclear supernatant (PNS) was prepared by a 6 min centrifugation at 1,100 *g*. PNS (950 μl) was loaded onto step gradient of 40, 35, 30, 25, 20 and 10% sucrose in the above lysis buffer.

The gradients were centrifuged at 4°C in SW40 rotor (Beckman) for 4.5 hr at 245,000 *g* with slow acceleration and deceleration. Twelve 1 mL fractions were collected from the top of the gradient, proteins were precipitated using 10% trichloroacetic acid and 10% of each pellet were analyzed by western blot.

#### Photobleach experiments

FRAP (Fluorescence Recovery After Photobleaching) studies were conducted on live NIH 3T3 cells expressing endogenous caveolin1-GFP. Cells were seeded in LabTek or Ibidi chambers 24 or 48 hr prior to experiment. Measurements were taken in growth media supplemented with 10 mM HEPES (Sigma), and the 37°C temperature was controlled by a heated stage incubator insert. FRAP experiments were performed on an inverted Zeiss LSM510 confocal microscope, using a 63 ×, 1.4 NA objective. Three frames were taken before photobleaching to determine the average pre-bleach fluorescence at starting point. A defined region of interest (ROI; 8 μm diameter) was photobleached at full laser power. Recovery of fluorescence was monitored by scanning the ROI at low laser power in movies taken at rate of one frame/2 or 3 s (120-180 frames/movie). The mean fluorescence intensity in the ROI and the mean non-cellular background were determined from the images using LSM510 software. After subtracting the background, the ROI fluorescence values were normalized to an unbleached region to correct for the loss in fluorescence caused by imaging. To be able to compare FRAP curves from different cells, the average fluorescence from 3 frames taken before photobleaching was set to 100% and the relative recovery in every cells was normalized to its initial level. 6-10 cells were imaged in each independent experiment.

#### Quantitative PCR

Total RNA was isolated from cells using the RNeasy Mini Kit (QIAGEN) and reverse transcribed using the High-Capacity RNA-to-cDNA Kit (Applied Biosystems). Quantitative PCR analysis of CAV1 was performed using the CAV1 TaqMan probe (Mm01129316_m1) and TaqMan Universal Master Mix II, with UNG (Applied Biosystems) on a ViiA7 Real-Time PCR System (Applied Biosystems). This was normalized against GAPDH (Mm99999915_g1).

#### Pulse-chase

Cells were depleted of methionine and cystine by incubating with DMEM without methionine or cystine (GIBCO) supplemented with dialysed FBS for 1 hr at 37°C. Cells were pulsed with 50 μCi/ml EasyTag EXPRESS ^35^S Protein Labeling Mix (Perkin Elmer) for 2 hr at 37°C, and washed into normal growth medium before chase for the times indicated. Cells were lysed in 1% Triton X-100 plus 1% octyl-glucoside, lysates were subjected to immunoprecipitation and samples were run on pre-cast 4%–20% Tris-Glycine gels. Gels were fixed in 10% acetic acid, then incubated with Amplify Fluorographic Reagent (GE Healthcare) and subsequently dried. Samples were exposed at −80°C on Fuji Super RX X-ray films and developed.

#### Cell stretching experiments

Cells were grown on fibronectin-coated deformable chambers to fit a ShellPa cell stretching device (Menicon Life Science). Cells were stretched for 60 min by 20% extension with cycles at 1.5Hz. For electron microscopy, cells were fixed while still undergoing stretch for 5 min, before removal of the chambers and further fixation. For membrane rupture assays the cell culture medium was supplemented with FITC-DEAE dextran 150KDa (Sigma) at 100 mg/ml, and NucRed Live 647 nuclear stain (Invitrogen) added before imaging.

For quantification of membrane rupture assays, maximum intensity projections were generated from z stacks acquired with a 20x objective. The FITC channel was subjected to thresholding so that signal from autofluorescence in non-ruptured cells was set to zero. All cells with uniform cytosolic FITC fluorescence were analyzed. Mean intensity in the FITC channel for individual cell areas was measured using ImageJ. Cells with mean pixel intensity < 10 were scored as not ruptured, cells with mean pixel intensity > 10 were scored as ruptured.

#### Cell tracker labeling

Cells were labeled with 10 μM CellTracker Red CMTPX (Invitrogen) in serum-free media for 45 min at 37°C then seeded as required.

#### Light microscopy

For indirect immunofluorescence, cells were rinsed in PBS, fixed in 4% PFA, blocked in 5% FBS supplemented with 0.1% Triton X-100 for permeabilization, and incubated with the appropriate antibodies. All confocal imaging was carried out using a Zeiss LSM510 inverted confocal microscope with a 63x, 1.4NA objective, driven by Zen software. TIR images were acquired using an Olympus TIR microscope equipped with 488, 546 and 647 nm lasers and fitted with a 100x, 1.45NA objective. STED images were acquired with a Leica SP8 gated STED microscope with a white light laser and 100x, 1.4NA objective. Pixel size for acquired STED images was set to 20 nm.

#### Electron microscopy

For counting of morphological caveolae, cells grown on MatTek glass bottomed Petri dishes were fixed in 2.5% glutaraldehyde, 2% paraformaldehyde in 0.1 M cacodylate buffer. Cells grown on fibronectin-coated silicon chambers for stretching were fixed while stretching by adding double strength fixative to an equal volume of culture medium for 5min, before removal of the chambers and exchanging to fresh normal strength fixative.

Cells were then processed on their growing substrate for EM: post fixed in 1% osmium tetroxide, dehydrated in an ascending ethanol series and embedded in CY212 resin. Cells were cut perpendicular to their growing substrate and ultrathin sections of cells were stained with 4% aqueous uranyl acetate and Reynolds lead citrate and viewed on a FEI Tecnai Spirit operated at 80kV. Quantification of caveolae were carried out by acquiring images to trace the outline of cells at 6500x magnification, assembling these images into complete profile of the cell using Adobe Photoshop, and then scoring all morphologically recognizable caveolae blind to the identity of the sample, with each caveolar bulb counted as one caveola in the case of clusters comprising multiple bulbs.

For immunolabeling, cells were grown on glass bottom Petri dishes (MatTek) or on fibronectin-coated silicon chambers, and fixed in 4% PFA in 0.1 M phosphate buffer pH 7.4 overnight at 4°C. For fixation while stretching, normal strength fixative was added to the cells while undergoing stretching and replaced with fresh fixative. After washing, cells were treated with either 0.1% sodium borohydride or 50 mM glycine in phosphate buffer for 15  min to block reactive aldehydes, and then permeabilized using 0.03% saponin in 20 mM phosphate buffer, 150 mM sodium chloride. Cells were incubated in normal goat serum (Aurion 905.002) for 40 min before incubation in either rabbit anti-caveolin1 antibody used at 1:200 or rabbit anti-GFP (Abcam ab6556) used at 1:100 for 4.5 hr at RT. After washing, cells were incubated in a 1:200 dilution of F(ab’)2 goat anti-rabbit ultrasmall gold (Aurion 100.166) overnight at 4°C. Cells were fixed with 2% glutaraldehyde in 0.1 M phosphate buffer for 30 min, washed with distilled water followed by silver enhancement of gold using R-Gent SE-EM (Aurion 500.033) reagent. Cells were then post fixed with 0.5% osmium tetroxide in water for 15 min on ice and processed for EM as above. Quantification was carried out by acquisition of 50 images at 6500x magnification, all selected to contain gold staining, and then assignation of gold-positive membranes to the different classes shown. In the case of clusters of multiple caveolae each distinct caveolar bulb counted as one caveola. All image analysis was blind to the identity of the samples.

For 3′3’-diaminobenzidine (DAB) staining of EHD1,2,4/mitochondrial APEX transfected cells, transiently transfected and non transfected cells grown on MatTek dishes were fixed in pre-chilled 2% glutaraldehyde in 0.1M cacodylate buffer plus 2 mM calcium chloride for 1 hr on ice. All subsequent steps were carried out on ice until resin infiltration. After 5 washes in buffer, cells were treated for 5min in buffer containing 20mM glycine to quench unreacted fixative, followed by several washes. Freshly prepared DAB (working concentration 0.5 mg/ml), prepared using free base DAB dissolved in 0.1M HCl plus hydrogen peroxide (0.03%) were mixed and filtered (0.2 μm) directly onto cells for 10min. The reaction was stopped by washing in buffer. Cells were post fixed in 1% osmium tetroxide for 1 hr followed by processing for EM as above. Cells containing DAB stained mitochondria identified by transmitted light were cut out from the discs of resin and mounted on dummy resin blocks with superglue. Ultrathin sections were cut parallel to the growing surface.

### Quantification and Statistical Analysis

N values are given in the Figure Legends, or are visible in the Figure as individual data points are all shown. In Figure 3B each data point is a single cell area. In Figure 4A each data point represents a complete reconstruction of the perimeter of a single cell, generated from 15-70 high resolution electron micrographs per cell. In Figure 4B each data point is generated by densitometric quantification of signal intensity of western blots from three separate experiments. In Figure 4D each data point is a measurement of the neck diameter of a single caveolar bulb, the data are pooled from three separate experiments. In Figures 4F and 4G the values shown are the frequency of different membrane morphologies identified in 50 electron micrographs containing positive staining. In Figure 4H each data point is a cluster of two or more caveolae immediately adjacent to each other and labeled with anti-caveolin1 antibodies. In Figure 6B each line is the mean of > 7 individual photobleached regions, and each line represents a separate experimental repeat. In Figure 6D each point is a separate biological replicate, itself based on four experimental replicates. In Figure 6F each data point represents the mean of densitometric measurements from three separate experiments. In Figure 7A each data point represents a complete reconstructions of the perimeter of a single cell, generated from 15-70 high resolution electron micrographs per cell. In Figure 7B each data point is a cluster of two or more caveolae immediately adjacent to each other and labeled with anti-caveolin1 antibodies. In Figure 7D and 7F each data point represents 6-10 images from a single experiment, each image containing 50-150 individual cells. Separate data points are separate experiments.

All statistical analysis was carried out using GraphPad Prism software. Details of statistical tests applied are given in the relevant Figure Legends, and the rationale for these tests was as follows. When two datasets are compared with each other as in Figure 3B a simple Student’s t test was applied. When multiple samples are compared with a single control dataset a one way ANOVA with Dunnett’s multiple comparisons test was employed to test whether each sample varies significantly from the mean. In the experiments shown in Figures 7D and 7F the values to be compared are paired, as they represent cells of different genotypes plated in the same mixed culture, and so a paired t test was employed to assess whether the differences between paired values are significant. Unpaired t tests were nonparametric and hence do not assume Gaussian distribution of the data. Other datasets were not assessed for Gaussian distribution.

## Author Contributions

I.Y., G.H., J.C., C.M.-T., E.S., and B.J.N. carried out experiments. C.G.H. provided reagents and preliminary observations. I.Y., E.S., and B.J.N. wrote the manuscript.

## Supplemental Information

Document S1. Figures S1–S7 and Table S1

Movie S1. TIR Images of Caveolin1-GFP Expressed by Genome Editing in WT and *ΔEHD1,2,3* NIH-3T3 Cells, Related to Figure 6A The movie plays at 15x real time.

Document S2. Article plus Supplemental Information
