# Supplementary material for: EHD Proteins Cooperate to Generate Caveolar Clusters and to Maintain Caveolae during Repeated Mechanical Stress
Source: Curr Biol. 2017 Oct 9;27(19):2951–2962.e5. doi: 10.1016/j.cub.2017.07.047 (PMC5640515; doi:10.1016/j.cub.2017.07.047)
Supplement: Document S1. Figures S1–S7 and Table S1 [file mmc1.pdf]

Current Biology, Volume *27*

Supplemental Information

EHD Proteins Cooperate to Generate Caveolar Clusters and to Maintain Caveolae during Repeated Mechanical Stress

Ivana Yeow, Gillian Howard, Jessica Chadwick, Carolina Mendoza-Topaz, Carsten G. Hansen, Benjamin J. Nichols, and Elena Shvets

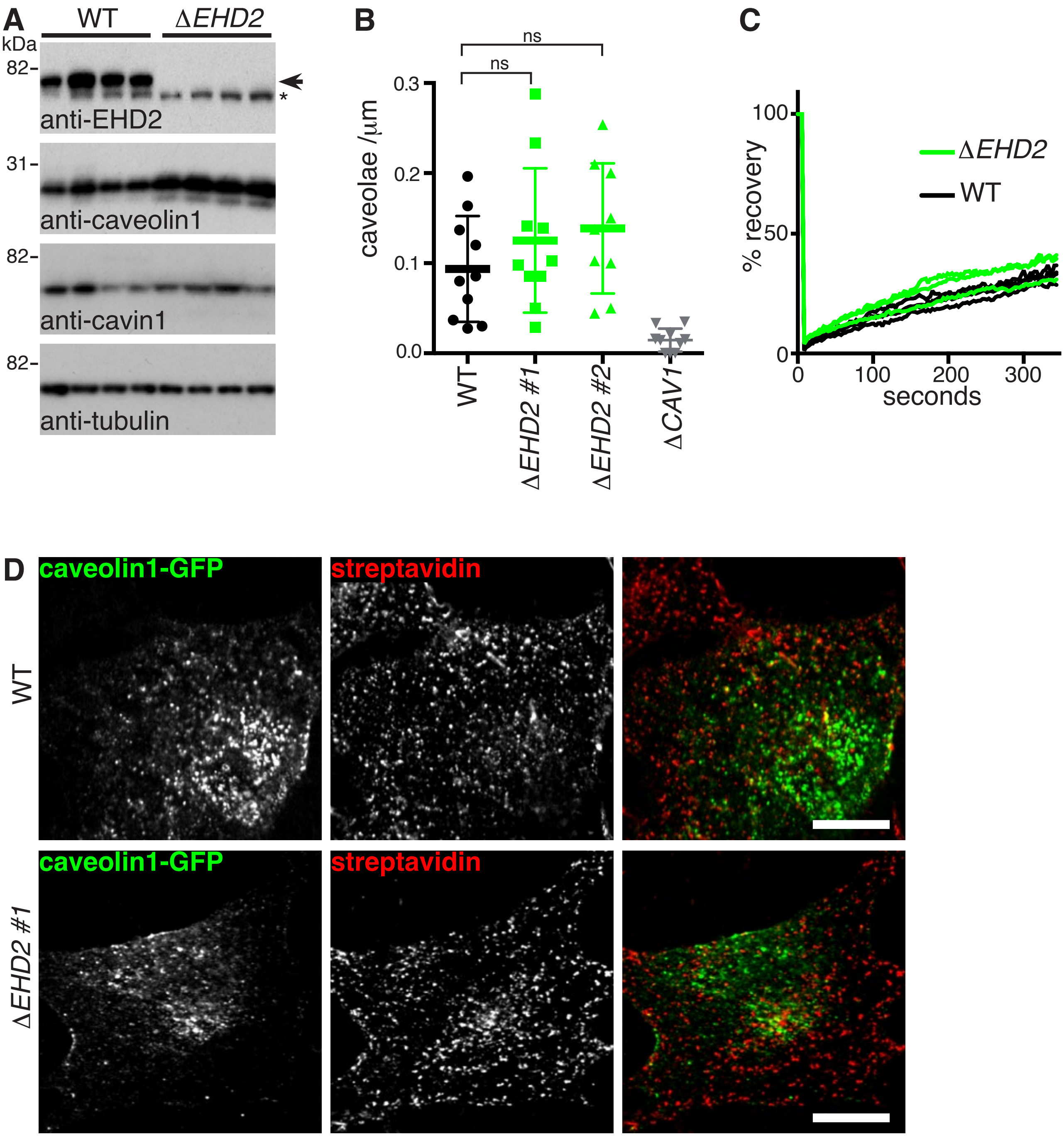

Figure S1. The abundance, morphology and dynamics of caveolae are not detectably altered in NIH 3T3 cells lacking EHD2, related to Figure 1. A. Western blots to show abundance of caveolar proteins in Δ*EHD2* cells. Blots from four cultures of wild-type NIH 3T3 cells and four clones of Δ*EHD2* cells derived from them are shown. Antibodies used in the different blots are shown. There is a background band detected with anti-EHD2 antibodies, indicated with a *, most likely due to cross-reaction with other EHD proteins. **B**. Quantification of morphologically-defined caveolae in knockout cells. For each genotype / clone complete reconstructions of the perimeter of 10 cells were generated from 15-70 high resolution micrographs per cell. Two different clones of Δ*EHD2* cells were analysed. Statistical analysis one-way ANOVA with Dunnett's multiple comparison test. **C**. Fluorescence recovery after photobleaching of caveolin1-GFP in wild-type NIH 3T3 cells and three different Δ*EHD2* clones. Each line is a mean from >7 individual photobleached regions. **D**. Internalisation assay to reveal intracellular caveolin1-GFP. Biotinylation of all surface proteins with sulfo-NHS-SS-biotin was followed by MESNA treatment to remove non-internalised biotin moieties and streptavidin-labelling to reveal endocytic compartments. Internalisation was for 15 min. Bar 10µm.

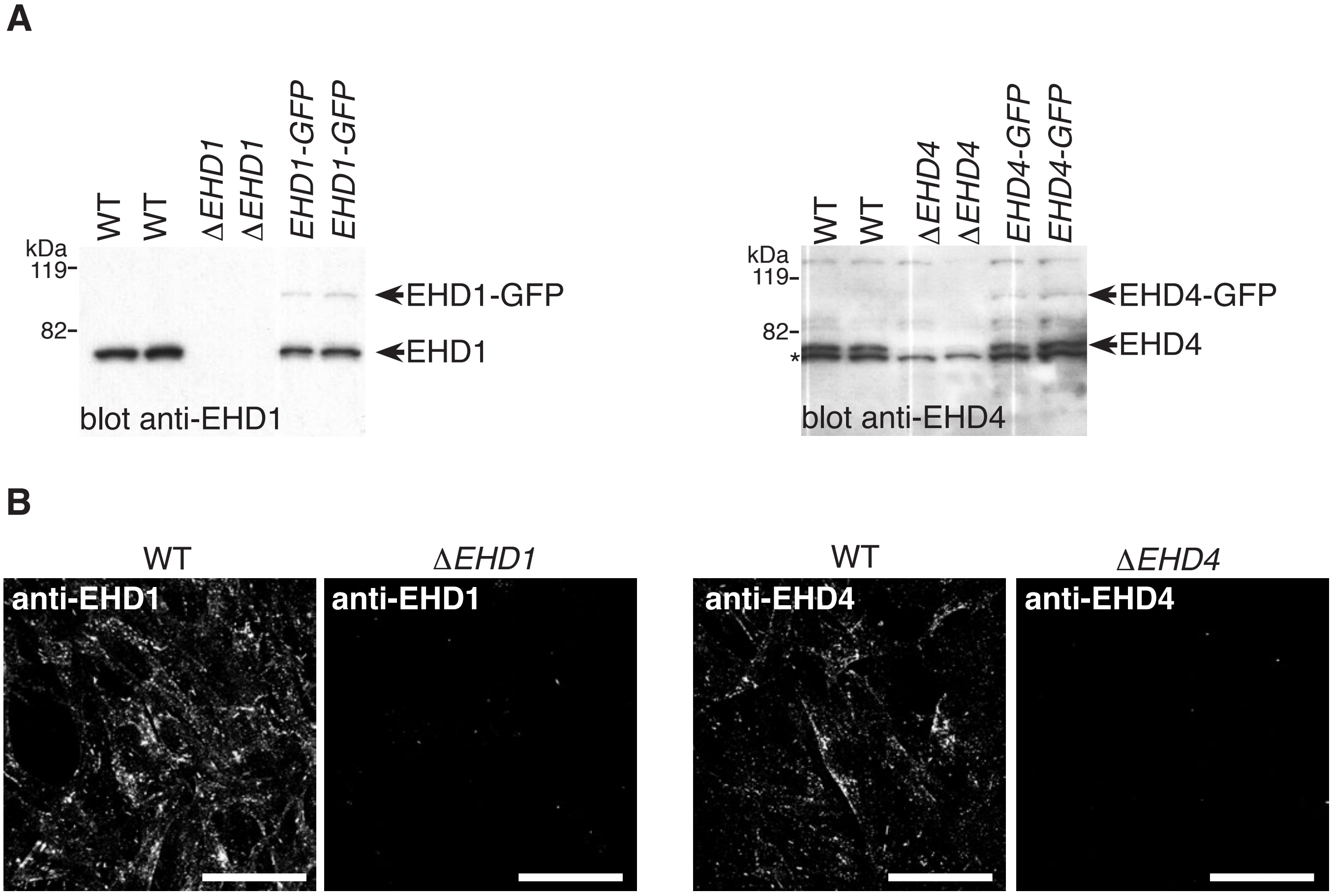

Figure S2. EHD1 and EHD4 knockout and knock-in cell lines, related to Figure 1. **A**. Western blots with the antibodies shown. Two different clones of the knockout cells, and knock-in cell lines are shown, along with two different cultures of the parental NIH 3T3 cells. Note that endogenous EHD1 and EHD4 are still detected in the EHD1-GFP and EHD4-GFP cell lines, so the GFP is unlikely to have been inserted into all *EHD1* and *EHD4* alleles. The * in the anti-EHD4 blot is a background band most likely generated by cross-reaction with other EHD proteins (Figure S6). **B**. Confocal images of Δ*EHD1* and Δ*EHD4* cells stained by indirect immunoflourescence with the antibodies shown. All images are of confluent fields of cells. Bars are 50μm.

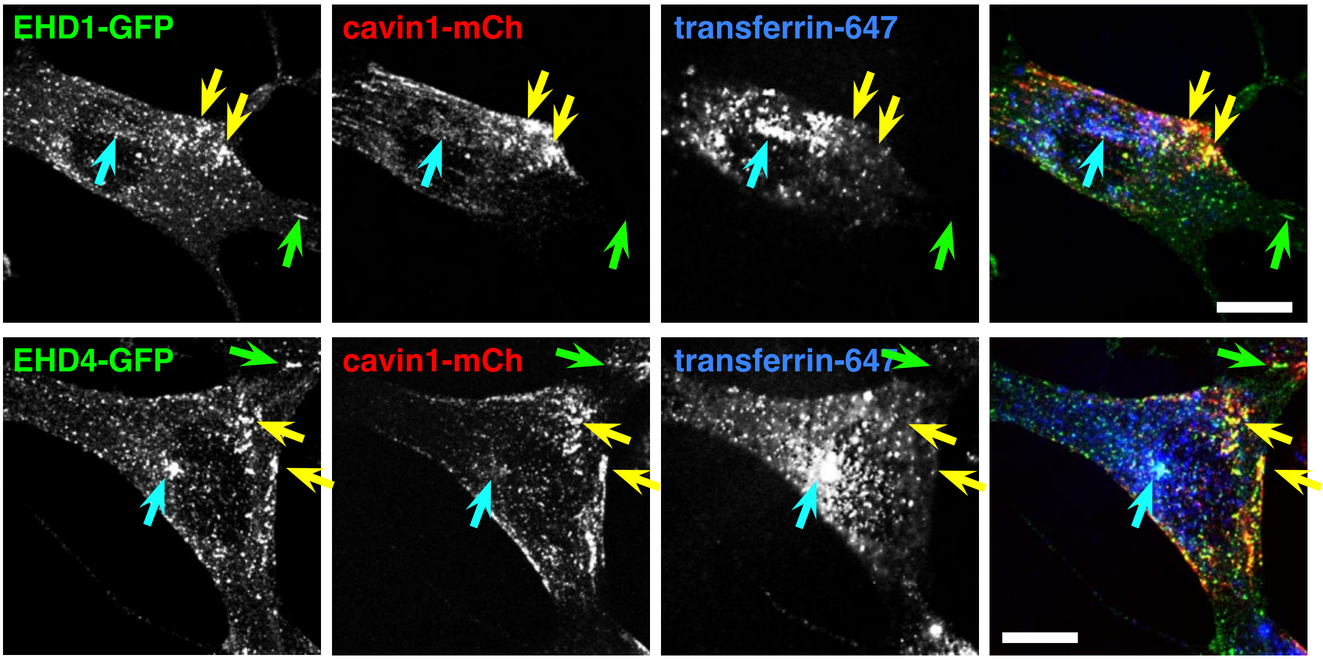

Figure S3. Confocal images to show overall cellular distribution of EHD1-GFP and EHD4-GFP, related to Figure 1. Cells were genome edited to express either EHD1-GFP or EHD4-GFP, and cavin1-mCherry. Cells were loaded with Alexa-647 transferrin for 30 min before fixation. Indirect immunofluorescence with anti-GFP antibodies was used to boost signals from the endogenously tagged proteins. Yellow arrows highlight co-localisation between EHD proteins and cavin1-mCherry, green arrows highlight linear presumptive tubes containing EHD proteins, and cyan arrows hightlight partial overlap between EHD proteins and endocytosed transferrin. Bar 10µm.

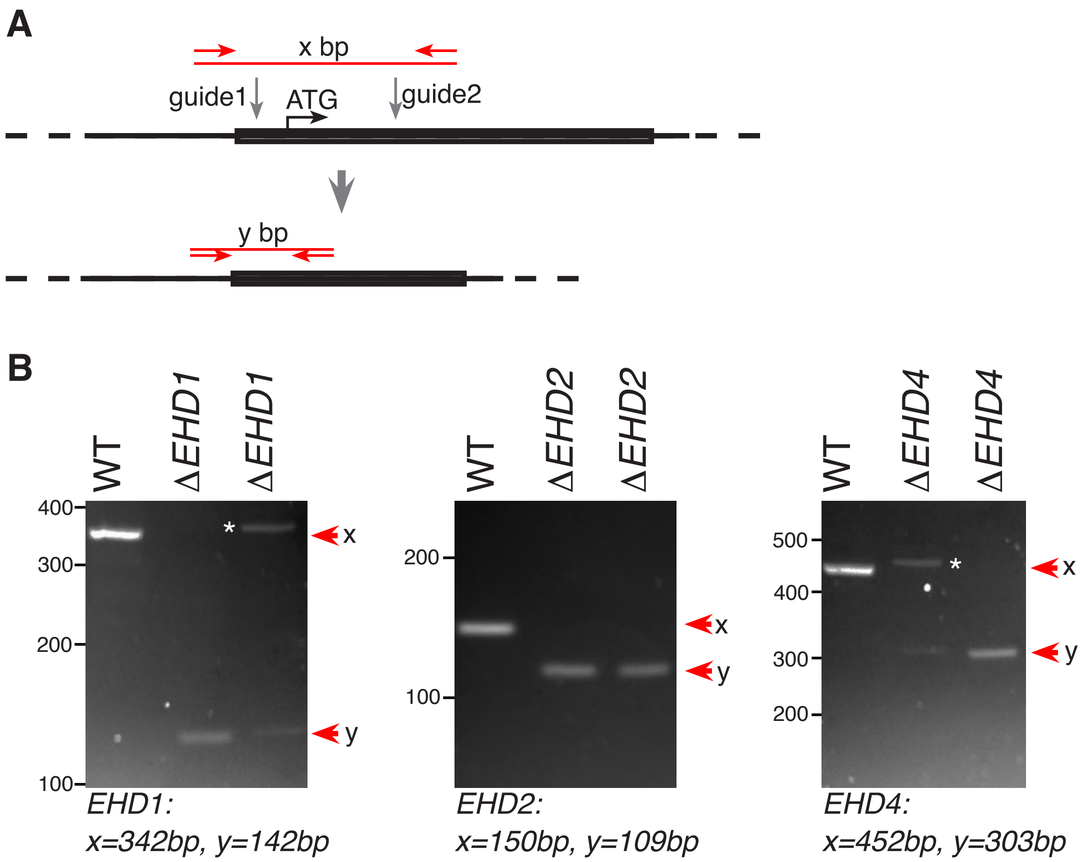

Figure S4. PCR genotyping of EHD knockout cell lines, related to Figure 3. A. Cartoon showing gene knockout strategy employing CRISPR with dual guide RNAs. The exon containing translation start ATG was targeted. Red lines show position PCR primers used for genotyping, producing products of x or y bp depending on genotype as shown. **B**. Agarose gel electrophoresis showing PCR products from genotyping as in A, with the predicted sizes of bands x and y. Asterisks show bands close to the size of band x in the knockout cells, which may result from cleavage of one allele with only one of the two guide RNAs used. In all cases no protein was detected by Western blotting, and sequencing of PCR products confirmed the presence of mutations.

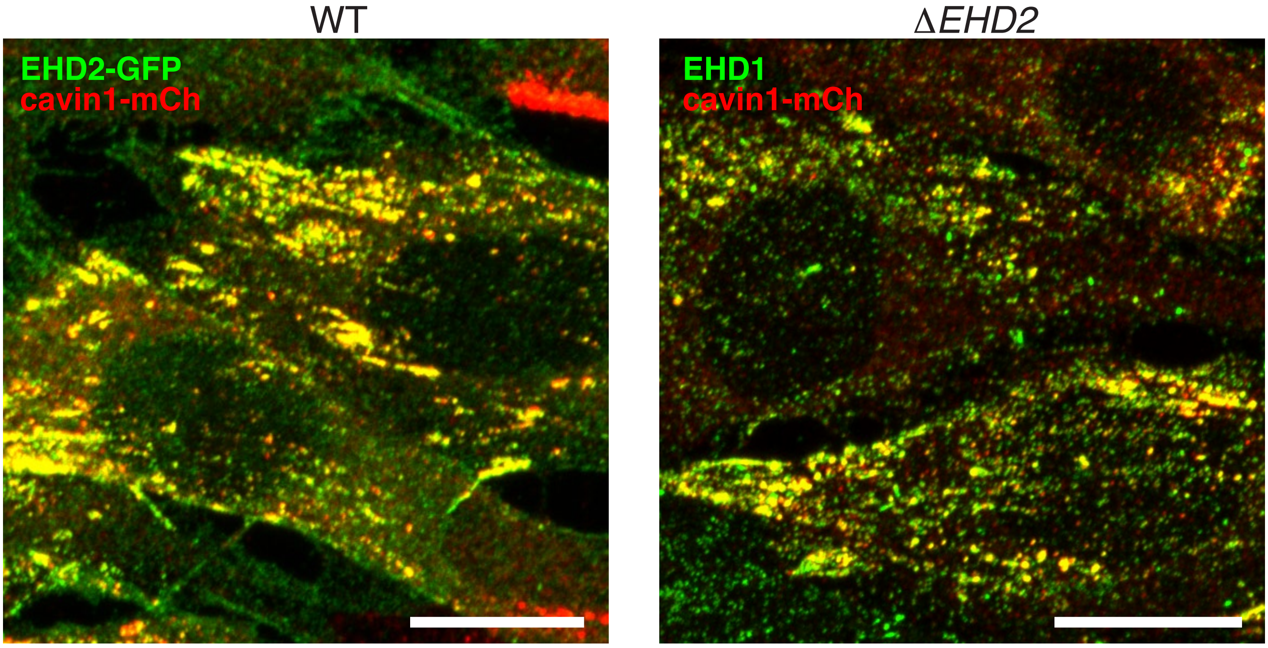

Figure S5. Confocal images to compare co-localisation between EHD2 and cavin1 in WT cells with that detected between EHD1 and cavin1 in Δ*EHD2* cells, related to Figure 3. Cavin1-mCherry was expressed by genome editing in both cell lines. EHD2-GFP was expressed by genome editing in the WT cells, while EHD1 was detected using specific antibodies and indirect immunofluorescence. Bar 10 µm.

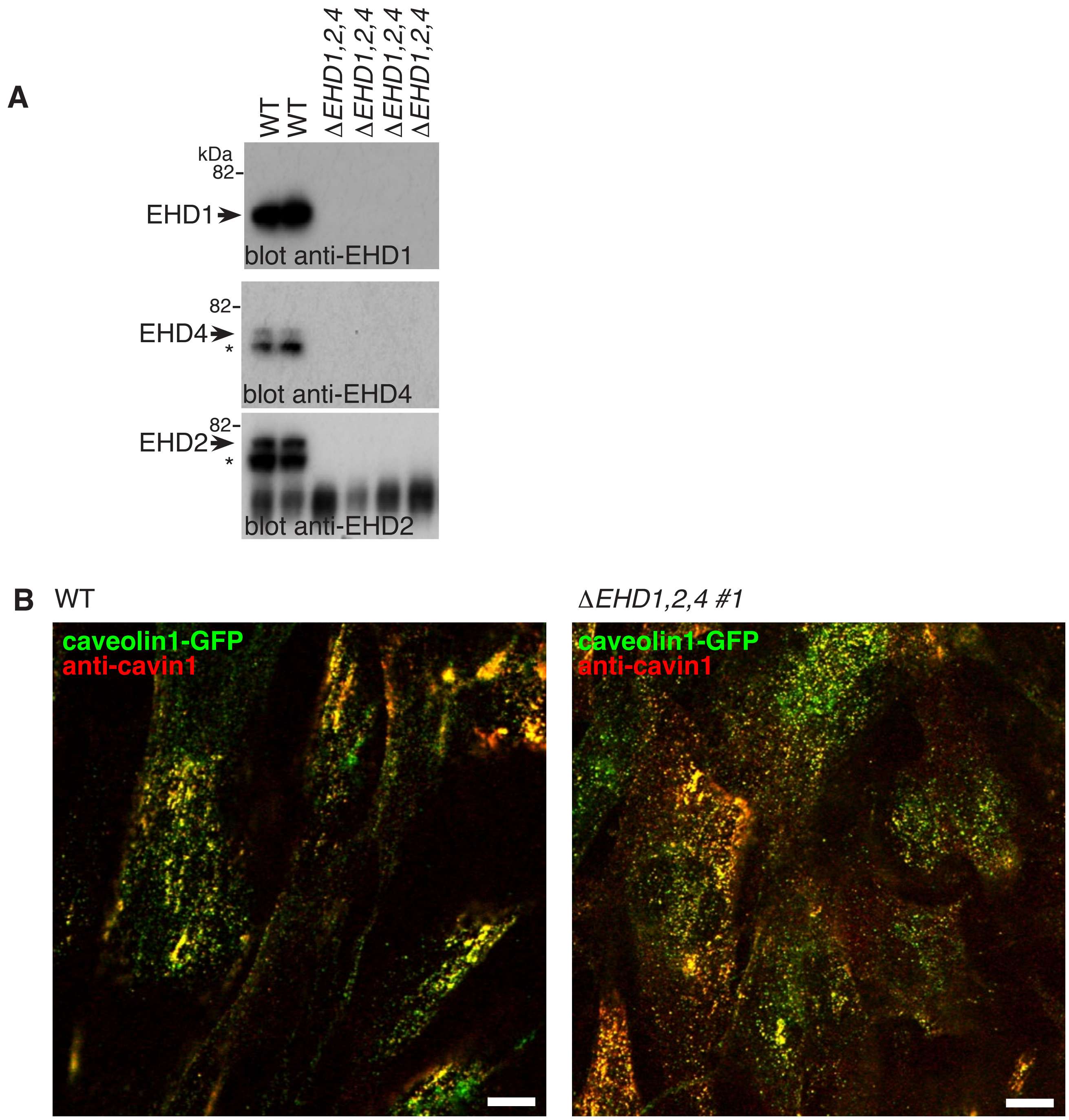

Figure S6. Δ*EHD1,2,4* cell lines, related to Figure 4. A. Four different clones are shown, along with two cultures of the parental NIH-3T3 cells. Blots were probed with the antibodies shown. Note that the lower bands in the WT anti-EHD4 and anti-EHD2 blots, marked *, persist in single knock-out Δ*EHD4* and Δ*EHD2* cells respectively, but are absent in the triple knockouts – they are therefore likely to represent cross-reaction of the antibodies with other EHDs. **B**. Confocal microscopy to show co-localisation between caveolin1-GFP and anti-cavin1 antibodies in Δ*EHD1,2,4* cells. NIH 3T3 cells expressing caveolin1-GFP by gene editing, and triple knockout cells expressing caveolin1-GFP in the same way, were fixed and stained with anti-cavin1 antibodies. Bar is 10µm.

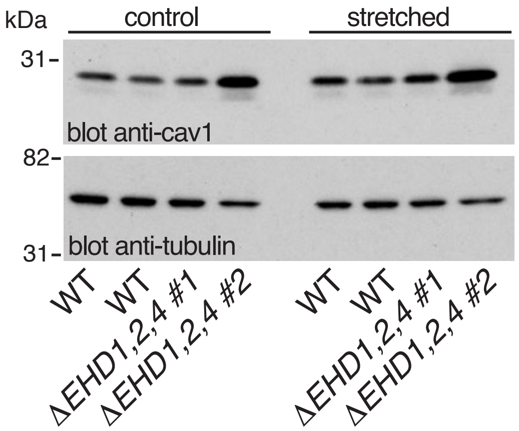

Figure S7. Expression of caveolin1 before and after stretching of cells for one hour, related to Figure 7. Representative Western blots are shown. Stretching was by 20% for 60min at 1.5Hz.

Oligo name	Sequence	Gene	Goal
EHD2 cleavage C-term pair	CACCGTTGGCTACACGGCTACACG<br>AAACCGTGTAGCCGTGTAGCCAAC	EHD2	Knock-in
EHD1 cleavage C-term pair	CACCGAAGTCACTCGTGCCTCCGTT<br>AAACAACGGAGGCACGAGTGACTTC	EHD1	Knock-in
EHD4 cleavage C-term pair	CACCGTGGGCCCTCAGTCAGCCTT<br>AAACAAGGCTGACTGAGGGCCCAC	EHD4	Knock-in
EHD2 cleavage N-term pair1	CACCGTGCGGATGGCCTCAGGTCGC<br>AAACGCGACCTGAGGCCATCCGCAC	EHD2	Knock-out
EHD2 cleavage N-term pair2	CACCGCGAAAGTCACCATGTTCAGC<br>AAACGCTGAACATGGTGACTTTCGC	EHD2	Knock-out
EHD1 cleavage N-term pair1	CACCGCAAGGATGCCCGCCGCAAGA<br>AAACTCTTGCGGCGGGCATCCTTGC	EHD1	Knock-out
EHD1 cleavage N-term pair2	CACCGCATCCGCCACCTGATCGAGC<br>AAACGCTCGATCAGGTGGCGGATGC	EHD1	Knock-out
EHD4 cleavage N-term pair1	CACCGCTGGCGGGCGCGAGCGCTC<br>AAACGAGCGCTCGCGCCCGCCAGC	EHD4	Knock-out
EHD4 cleavage N-term pair2	CACCGCTGGTGGGCCAGTACAGCA<br>AAACTGCTGTACTGGCCCACCAGC	EHD4	Knock-out
Cav1 cleavage N-term pair1	CACCGGGCAAATACGTAGACTCCG<br>AAACCGGAGTCTACGTATTTGCCC	Caveolin1	Knock-out
Cav1 cleavage N-term pair2	CACCGAGTGTATGACGCGCACACCA<br>AAACTGGTGTGCGCGTCATACACTC	Caveolin1	Knock-out
Left arm for	GGATAATGCAACTCGTGCTTAAAG	EHD2	Knock-in
Left arm rev	CGGGCCCGCGGTACCGTCGACTGCAGAATTTTCAGCAGAGCCCTTCTGTC	EHD2	Knock-in
Right arm for	GACAGCAGCGACTGCTGG	EHD2	Knock-in
Right arm rev	CAATGCCCCAGGATATCCATG	EHD2	Knock-in
Left arm for	AGGGGTTCCGCGCACATTTCCCCTCGAGTGGTGAACAACCTGGGAGAG	EHD1	Knock-in
Left arm rev	CGGGCCCGCGGTACCGTCGACTGCAGAATTCTCGTGCCTCCGTTTGGAG	EHD1	Knock-in
Right arm for	TCTCGGCATGGACGAGCTGTACAAGTAACTTCCATGCCTGAGATACCC	EHD1	Knock-in
Right arm rev	GATAACCGTATTACCGCCATGGCGGCCGCCAGGTCACAGGGCCTACTG	EHD1	Knock-in
Left arm for	ATAGGGGTTCCGCGCACATTTCCCCTCGAGAGCAGATGTGGCCCCTCG	EHD4	Knock-in
Left arm rev	CGGGCCCGCGGTACCGTCGACTGCAGAATTGTCAGCCTTTGGCAGGGAC	EHD4	Knock-in
Right arm for	TCACTCTCGGCATGGACGAGCTGTACAAGTAAGGGCCCACAGCTGGGG	EHD4	Knock-in
Right arm rev	GATAACCGTATTACCGCCATGGCGGCCGCGCCCAGATAGGCACTCCTG	EHD4	Knock-in
GFP for	AATTCTGCAGTCGACGGTACCGCGGGCCCGATGGTGAGCAAGGGCGAGG	GFP (for EHD2)	Knock-in
GFP rev	CCAGCAGTCGCTGCTGTCTTACTTGTACAGCTCGTCCATG	GFP (for EHD2)	Knock-in
GFP for	AATTCTGCAGTCGACGGTACCGCGGGCCCGATGGTGAGCAAGGGCGAGG	GFP (for EHD1 and 4)	Knock-in
GFP rev	TTACTTGTACAGCTCGTCCATG	GFP (for EHD1 and 4)	Knock-in

Table S1. Oligonucleotide sequences used for generating knockout and knock-in cell lines, related to STAR Methods. EHD1, 2 and 4 cleavage C-term pair were used for insertion into pSpCas9(BB)-2A-Puro (PX459) to target the stop codon for cleavage. This was used with donor DNA for knock-in of a fluorescent tag at the C-terminal. Right/Left arm for/rev primers were used to amplify ~1 kb of genomic DNA on either side of the gene stop codon for donor DNA flanking regions. DNA coding for GFP was amplified with GFP for/rev. Donor DNA constructs were produced with the above left flanking region, fluorescent protein DNA fused to a linker and right flanking region. EHD1, 2 and 4 and Cav1 cleavage N-term pair were inserted into pSpCas9(BB)-2A-GFP (PX458) for generation of KO cells. Pair1 and pair2 for each gene was used to cleave out ~50-200 bp of genomic DNA at the start codon.
