# Supplementary figures and images for: EHD Proteins Cooperate to Generate Caveolar Clusters and to Maintain Caveolae during Repeated Mechanical Stress
Source: Curr Biol. 2017 Oct 9;27(19):2951–2962.e5. doi: 10.1016/j.cub.2017.07.047 (PMC5640515; doi:10.1016/j.cub.2017.07.047)

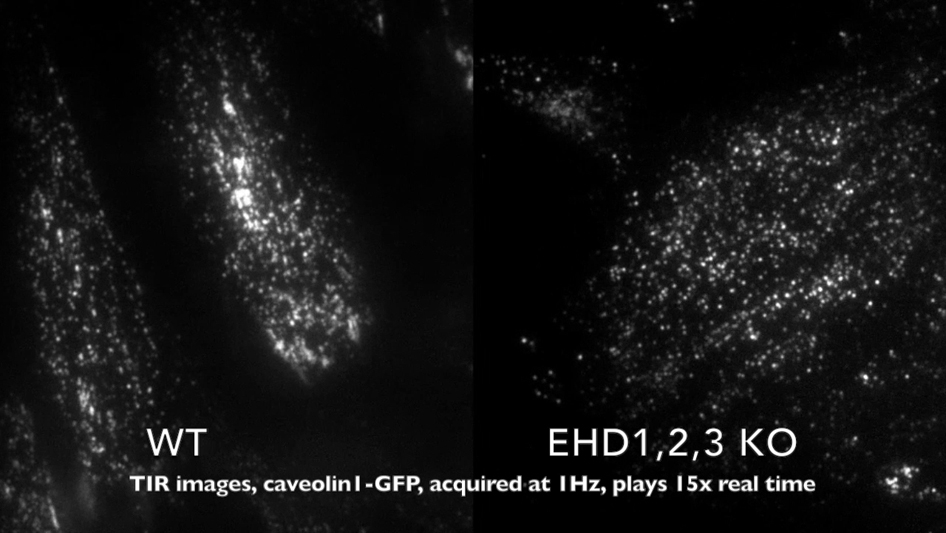

Supplement: Movie S1. TIR Images of Caveolin1-GFP Expressed by Genome Editing in WT and ΔEHD1,2,3 NIH-3T3 Cells, Related to Figure 6A — The movie plays at 15x real time. [file mmc2.jpg]
